# Dynamic Structure and Stability of DNA Duplexes Bearing a Dinuclear Hg(II)-Mediated Base Pair

**DOI:** 10.3390/molecules25214942

**Published:** 2020-10-26

**Authors:** Jim Bachmann, Isabell Schönrath, Jens Müller, Nikos L. Doltsinis

**Affiliations:** 1 Institute for Solid State Theory and Center for Multiscale Theory and Computation, Westfälische-Wilhelms Universität Münster, Wilhelm-Klemm-Straße 10, 48149 Münster, Germany; j_bach04@uni-muenster.de; 2 Institut für Anorganische und Analytische Chemie, Westfälische-Wilhelms Universität Münster, Corrensstraße 30, 48149 Münster, Germany; isabell.schoenrath@uni-muenster.de

**Keywords:** Ab initio calculations, DNA structures, molecular dynamics, free energy, mercury, Hg(II), metal-mediated base pairs, metal-modified DNA

## Abstract

Quantum mechanical (QM) and hybrid quantum mechanical/molecular mechanical (QM/MM) molecular dynamics simulations of a recently reported dinuclear mercury(II)-mediated base pair were performed aiming to analyse its intramolecular bonding pattern, its stability, and to obtain clues on the mechanism of the incorporation of mercury(II) into the DNA. The dynamic distance constraint was employed to find initial structures, control the dissociation process in an unbiased fashion and to determine the free energy required. A strong influence of the exocyclic carbonyl or amino groups of neighbouring base pairs on both the bonding pattern and the mechanism of incorporation was observed. During the dissociation simulation, an amino group of an adenine moiety of the adjacent base pair acts as a turnstile to rotate the mercury(II) ion out of the DNA core region. The calculations provide an important insight into the mechanism of formation of this dinuclear metal-mediated base pair and indicate that the exact location of a transition metal ion in a metal-mediated base pair may be more ambiguous than derived from simple model building.

## 1. Introduction

Nucleic acids with metal-mediated base pairs have been of interest in the development of functional nanostructures [1] as they feature robustness, programmable hybridization properties and a well-established automated synthesis [2,3]. Metal-mediated base pairs provide a convenient means for the site-specific functionalization of nucleic acids with metal ions. They can be formed from canonical nucleobases, with thymine and cytosine being the most prominent natural nucleobases capable of engaging in stable metal-mediated base pairs [4,5,6]. In this regard, thymine is known for its high affinity for mercury(II), whereas cytosine prefers binding to silver(I) [7,8,9]. In addition, many synthetic nucleobase analogues have been devised, broadening the metal-ion scope of metal-mediated base pairing. As a result, metal-mediated base pairs are known involving Cu(II), [10,11,12,13] Cu(I), [14] Mn(III) [15], Ni(II) [16], Zn(II) [17], and other metal ions [18,19]. Nevertheless the most prominent metal ions used with artificial nucleobases remain Ag(I) and Hg(II), too [20,21,22,23,24,25,26,27]. Nucleic acids comprising metal-mediated base pairs have been applied in various manners, e.g., in the context of modulating the charge transfer capability of the nucleic acids [28,29,30,31,32], in metal-responsive structural transformations [33,34,35], in the formation of DNA-templated silver nanoclusters [36], and in oligonucleotide recognition [37,38,39]. Various experimental structures have been reported, confirming that the concept of metal-mediated base pairing is compatible with various DNA duplex topologies [40]. Interestingly, metal-mediated base pairs can even be introduced into nucleic acids enzymatically [41,42,43,44,45,46,47].

Depending on the type of artificial nucleobase used, even di- and trinuclear metal-mediated base pairs have been reported [48,49,50,51]. Recently, the first dinuclear metal-mediated base pair containing two mercury(II) ions as divalent metal ions has been reported [52]. The artificial nucleobase 1,*N*^6^-ethenoadenine (εA) had been employed in those studies because it had been shown to provide a unique coordination mode, with the lone pairs of its two coordinating nitrogen atoms being oriented in an almost parallel fashion. As a result, εA is particularly suited for binding two metal ions at close distance to each other. Hence, the use of εA allowed for the first time the arrangement of two Hg(II) ions (bearing four positive charges) in a single metal-mediated base pairs. The bonding pattern of the resulting εA–Hg(II)_2_–T base pair has been explored at the Density Functional Theory (DFT) level for isolated base pairs [52]. Later, a slightly different geometry has been proposed for this base pair [53]. As mercury features a multitude of possible bonding patterns within DNA [54], we decided to study the bonding within this mercury(II)-modified DNA by the means of QM and QM/MM simulations. As no empirical structures of the solvated DNA duplex were available, we employed the so-called dynamic distance constraint [55] to find an initial geometry of the DNA in the preferred hydrogen bonding pattern. After this initial phase, all constraints are released for a further optimization, which preserved the hydrogen bonding structure.

Free energy simulations are performed to study the stability and possible dissociation paths of the novel DNA, where the neighbouring base pairs influence both the bonding pattern and the inclusion path of mercury(II) into the DNA.

## 2. Computational Details

### 2.1. Static DFT Calculations

The GAUSSIAN09 software [56] was utilized using density functional theory (DFT) employing the PBE0 hybrid-functional [57,58,59,60] and the SDD basis set [61,62] together with Grimme D3 corrections with Becke–Johnson damping [63,64] and the PCM continuum solvation model [65].For convergence criteria the Gaussian *Tight* option was chosen.

The influence of relativistic effects and different exchange-correlation functionals was investigated using the ORCA 3.0.3 software [66]. The functionals PBE, PBE0, BP86, PM6, BLYP and B3LYP were chosen with basis sets TZVP and SDD. Scalar relativistic ZORA corrections [67] were evaluated using special scalar relativistic basis sets [68]. As convergence criteria the *NormalSCF* criteria of ORCA were set.

### 2.2. Topology Generation for Classical Molecular Dynamics

Using the program tleap from the AMBERTOOLS package [69], the PARMbsc1 force field [70] specially refined for DNA simulations and Hg van der Waals parameters devised by Fuchs et al. [71], a topology was generated for the DNA double helix. A parametrization of isoguanine was not available, so a force field was parametrized with RESP charges [72], bond lengths and angles from the DFT/PBE0/SDD optimization of the isolated base pair and bond strength parameters from the guanine parameters of PARMbsc1. The thus prepared DNA was then solvated by tleap in a 12 Å thick water shell to screen self-interactions. This resulted in an orthorhombic box with side lengths 44.41 Å, 44.57 Å and 63.41 Å including 3073 water molecules and 23 Na^+^ counter ions, neutralizing 26 negative charges due to the phosphate backbone and the three positive charges of the modified base pair, leading to an overall charge-neutral system.

For the isolated base pair, a new set of partial atomic charges was generated from a GAUSSIAN09 DFT calculation with the SDD basis and the PBE0 functional, Grimme-D3 corrections, PCM implicit solvation and the CHelpG method, which assigns RESP charges to each atom. 

### 2.3. Classical Molecular Dynamics

Classical molecular dynamics (MD) simulations were performed using the FIST method of CP2K [73] with the PARMbsc1 force field [70] for the DNA and the Na^+^ counterions, with the modifications for isoguanine described above. Water molecules were described by the TIP3P water model [74,75]. A time step of 0.5 fs was chosen. Equilibration runs were performed in the *NPT* ensemble using the Nose–Hoover barostat and thermostat with a chain length of 3 and a time constant of 1 ps. The target temperature was set to 300 K in all *NVT* and *NPT* runs and the target pressure to 1 bar in all *NPT* MDs.

### 2.4. Ab Initio Molecular Dynamics and Optimization

DFT based ab initio molecular dynamics simulations were carried out with the CP2K program using the GAPW scheme [76,77]. The PBE functional was chosen together with the TZVP-MOLOPT basis set for all atoms except mercury, for which a DZVP-MOLOPT basis was utilized [78], in conjunction with Goedecker–Teter–Hutter pseudopotentials [79,80,81] and a plane wave cut-off of 400 Ry. Grimme D3 dispersion corrections were employed with Becke–Johnson damping [63,64]. A total of 700 virtual molecular orbitals were calculated for Fermi-Dirac smearing due to the presence of the transition metal mercury. Cholesky inversion was chosen as the solver of the eigenvalue problem, while Broyden mixing was employed for optimization. MD was performed with a time step of 0.5 fs. 

The convergence criteria for the geometry optimization were set to 0.003 a.u. for the maximum step size, 0.0015 a.u. for the root-mean-square (RMS) displacement of atomic positions, 0.0004 a.u. for gradients and 0.003 a.u. for the RMS change of the gradients. 

### 2.5. QM/MM Simulations

**Figure 1:**
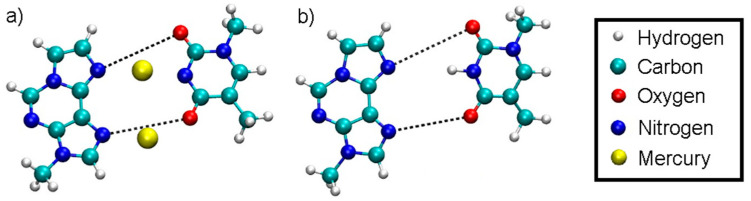
Definition of the dynamic distance within the plane of the modified base pair with Hg (**a**) and without Hg (**b**). The dynamic distance within the plane of the isolated base pair is defined in the same way as for the complete DNA duplex.

QM/MM molecular dynamics simulations were carried out with the CP2K program, where for the classical region the settings from 2.3 were applied. For the QM region, settings from Section 2.4 were used. The QM region consisted of all atoms of the metal-modified base pair (Figure 1a) and the base pairs directly above and below. Furthermore, three negatively charged phosphate groups together with the attached backbone structure were incorporated into the QM subsystem to neutralize the threefold positive charge of the metal-modified base pair leading to an overall charge-neutral QM subsystem with multiplicity 1, which proved crucial for obtaining reasonable geometries and stable MD simulations. Coupling was treated with the Gaussian expansion of electrostatic potentials (GEEP) method in CP2K, where the IMOMM method was utilized to treat the links between MM and QM [82].

### 2.6. Free Energy Calculations

#### 2.6.1. Dynamic Distance Constraint

Five different model systems termed structure **1**–**5**, which will be presented in Section 3, were investigated by free energy calculations.

As a reaction coordinate, we chose the dynamic distance [55].

(1) $$D={\left(\underset{\mathrm{NOP}}{\sum }\frac{\mu_{ij}}{\mu^{*}}{\left(r_{i}-r_{j}\right)}^{2}\right)}^{\frac{1}{2}}$$

where NOP are all non-overlapping pairs *i*, *j* of atoms between which the constraint is defined, $r_{i}$ and $r_{j}$ are their positions, $\mu_{ij}=m_{i}m_{j}/\left(m_{i}+m_{j}\right)$ is their reduced mass and $\mu^{*}=\sum_{NOP}\mu_{ij}$ is the sum of all reduced masses.

In the case of the full DNA models *D* = *D*_DNA_ includes all N···H hydrogen bonds within regular base pairs and the two N···O distances bridged by Hg(II) ions (Figure 1). In the case of the isolated base-pair models, the two O···N distances according to Figure 1 were selected to enter the dynamic distance *D* = *D*_ISO_.

#### 2.6.2. Thermodynamic Integration

The dynamic distance was incrementally increased and the average Lagrange multiplier at every constraint value, corresponding to the average constraint force <*F*(*D*)>, calculated. Integrating over the mean constraint force along the dynamic distance gives the free energy necessary to dissociate the system:

(2) $$∆A=\;\int_{D_{min}}^{D_{max}}\langle F\left(D\right)\rangle \;dD$$

Structures **1**, **2**, and **3** were simulated for 500 fs at the initial dynamic distance before starting the dissociation MD, to allow for further equilibration of the quantum region. The running average of the Lagrange multipliers are shown in Figure S4 for structure **1**, Figure S5 for structure **2** and Figure S6 for structure **3**. For the calculations involving structure **1_,_** the dynamic distance was initially set to 3.0 Å and increased/decreased by 0.2 Å at each simulation point, where each point was simulated for at least 1 ps. Overall 41360 simulation steps were performed for structure **1_,_** equalling 20.58 ps. At the final point of *D*_DNA_ = 5.8 Å of the trajectory, the modified base pair was found to be dissociated and one Hg(II) ion was in contact with water. Hence, the simulation was not continued further as this would have led to unphysical water-mercury interactions across the QM/MM boundary. Structure **2** was less stable during the dissociation simulation, thus requiring smaller dynamic distance steps of 0.1 Å. The system was simulated from an initial value of *D*_DNA_ = 3.7 Å up to a final value of *D*_DNA_ = 10.5 Å, at which point only a change in the bonding pattern occurred instead of dissociation. However, as one mercury ion was already in contact with MM water at this *D*_DNA_, the simulation was not continued further. Each *D* point was simulated for at least 1 ps, amounting to an overall simulation length of 62 ps. Structure **3** was simulated with a dynamic distance step size of 0.1 Å from *D*_DNA_ = 2.5 Å to *D*_DNA_ = 3.5 Å and a step size of 0.5 Å for the remaining points up to *D*_DNA_ = 8.5 Å. The overall simulation length for structure **3** was about 35 ps, after which the modified base pair was sufficiently dissociated (see Figure S3).

In the all-QM simulations, structures **4** and **5** were dissociated by an increase of the dynamic distance between the N and O atoms of the modified base pair (Figure 8), termed *D*_ISO_, with settings given in Section 2.4. The system was simulated for 500 fs at the initial value of the dynamic distance constraint, which was subsequently increased in steps of 0.2 Å. An all-QM MD was performed with the CP2k code, with identical settings as before but with all water molecules described by DFT. A total of ca. 6 ps were simulated for structure **4** and ca. 13 ps for structure **5**.

## 3. Model Structures

In total, five different model structures were simulated, three of which were complete DNA models and two were isolated base pairs in solution. Their generation will be described below.

### 3.1. Generation of the Initial DNA Geometry

**Figure 2:**
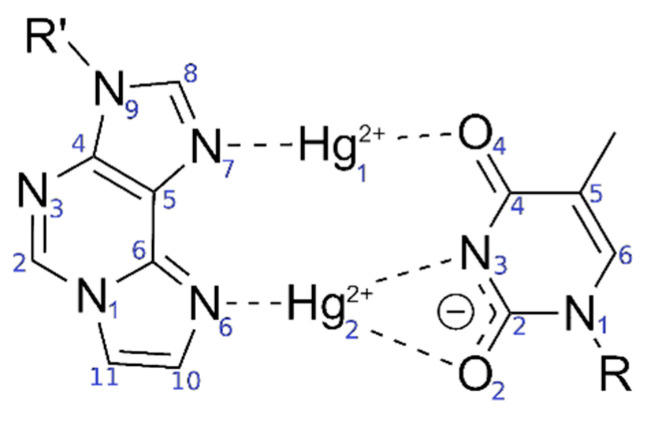
Originally proposed [52] bonding pattern of the dinuclear mercury(II)-mediated base pair between 1,*N*^6^-ethenoadenine (**εA**) and thymine (**T**), including the atom numbering according to IUPAC recommendations in blue.

**Figure 3:**
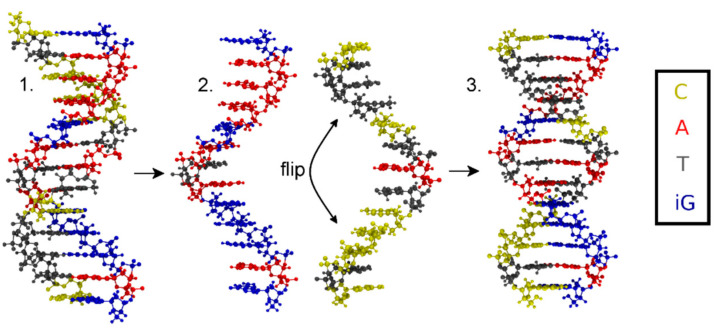
Flowchart depicting the generation of the initial structure with (iso)guanine residues depicted in blue, adenine in red, thymine in grey and cytosine in yellow. **1**. Antiparallel-stranded DNA obtained from 3DNA; **2**. Separated strands, where one stand is flipped by 180°; **3**. Reattached strands forming parallel-stranded DNA.

Five different initial structures are simulated by means of molecular dynamics and geometry optimizations to study the bonding patterns of the novel artificial **T:εA** base-pair (Figure 2). As no experimental structures of this mercury(II)-containing DNA duplex are available, the initial geometries were generated by using the 3DNA software [83] for the sequence **3′-d(GAAAGATAGGGAG)-5′/5′-d(CTCCCTATCTTTC)-3′**. One DNA strand was then rotated by 180° and placed back into a helical structure, to generate parallel-stranded DNA as used in the previous experiments (Figure 3). This was followed by an exchange of the exocyclic O and NH_2_ groups of all guanines, transforming them into isoguanines, thus generating the duplex **5′-(^i^GA^i^G^i^G^i^GATA^i^GAAA^i^G)-3′/5′-d(CTCCCTATCTTTC)-3′** corresponding to the one used in the original experiments [52] except for the modification of the central **A:T** base pair.

**Figure 4:**
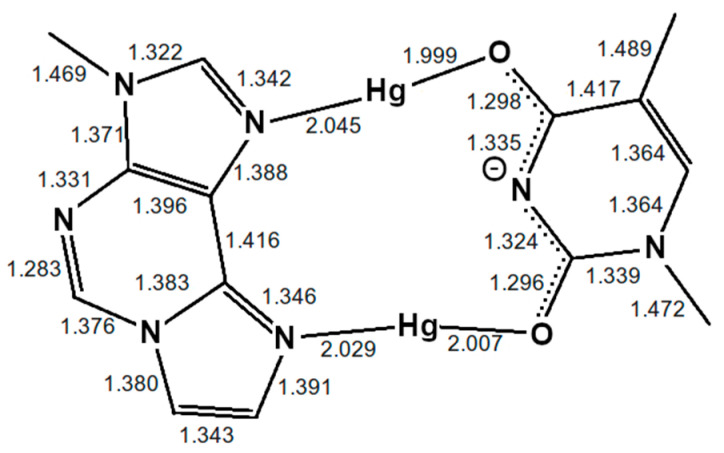
Optimized structure of the **εA**–Hg(II)_2_–**T** base pair with interatomic distances in Å. The N3 atom of thymine is deprotonated. Both mercury(II) ions form coordinate bonds involving one nitrogen atom of **εA** (N6 or N7) and one oxygen atom of **T** (O2 or O4), resulting in the **NO**-bonding pattern.

Only standard bases are available in the 3DNA program; thus, a thymine:adenine (**T**:**A**) base pair was generated at the site of the modified base pair. The atomic vacuum structure of the modified thymine:1,*N*^6^-ethenoadenine (**T**:**εA**) pair was optimized using the settings from Section 2.1. The electric charge of the base pair is +3, its spin multiplicity 1. The resulting structure is shown in Figure 4. This structure closely resembles that reported in reference 53 for this base pair.

**Figure 5:**
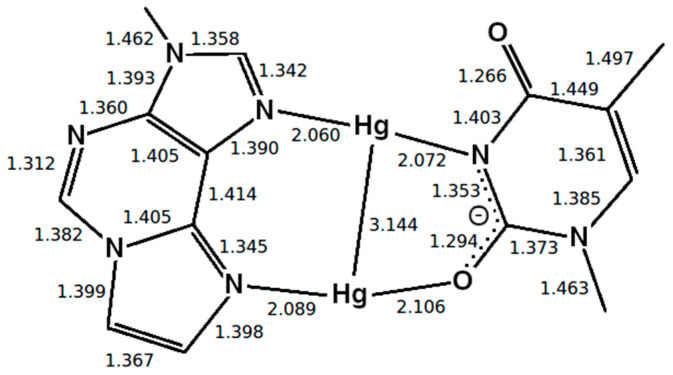
Optimized structure of the **εA** –Hg(II)_2_–**T** base pair with interatomic distances in Å, where the presence of neighbouring base pairs was taken into consideration, too. Only the metal-mediated base pair is shown. The mercury(II) previously attached to **T**-O4 changes its bonding pattern, now being located between two nitrogen atoms (**NN**-bonding pattern).

To assess the influence of the neighbouring base pairs onto the equilibrium geometry of the artificial base pair, further DFT geometry optimizations including the base pairs above and below **T**: **εA** have been performed with identical functionals and basis set as the metal-mediated base pair alone. In this model, adjacent base pairs are connected via a phosphate backbone. Due to the four phosphate moieties connecting the three base pairs, the charge of the system is now −1 and the multiplicity 1. Figure 5 displays the resulting structure. This structure is more similar to the originally proposed base pair geometry from reference 52.

Two different bonding patterns emerged during the geometry optimization. Within the isolated base pair, the mercury ion Hg2 is located in between the **εA**-N6 and **T**-O2 atoms, while Hg1 coordinates to the **εA**-N7 and **T**-O4 atoms (**NO** bonding pattern, Figure 4). When the adjacent pairs are also taken into account, Hg2 remains located between the **εA**-N6 and **T**-O2 atoms, while Hg1 changes its bonding pattern and is now coordinated to **εA**-N7 and **T**-N3 (**NN** bonding pattern, Figure 5). These two structures correspond to two minima on the potential energy surface and exist for both the isolated and the stacked base pair. In the isolated case, the **NO** bonding pattern represents the global minimum structure. This has been confirmed for a number of different exchange-correlation functionals given in Section 2.1; an overview of the results is tabulated in Figure S7. In the three base pair stack, the **NN** bonding pattern is favoured (Figure 5), where the neighbouring N and O atoms from the nucleobases above and below attract the mercury ions, if they are situated directly on top or below them.

As the PBE functional correctly predicts the **NO** pattern to be the more stable configuration for the isolated base pair, it was chosen as the exchange-correlation functional for the QM/MM and the all-QM MD runs in view of its computational advantage compared with hybrid functionals. Relativistic corrections did not change the observed bonding pattern (see Figure S7) and were thus not included in the MD runs. Because the TZVP basis led to a further stabilization of the **NO** compared to the **NO** pattern, the TZVP-MOLOPT basis was chosen for use during the MD.

**Figure 6:**
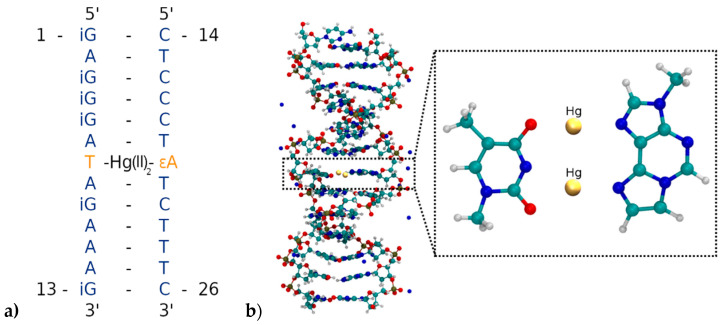
(**a**) Parallel-stranded DNA duplex with numbering of the nucleobases from the 5′ to the 3′ end. **T**7 is deprotonated and forms a dinuclear Hg(II)-mediated base pair with the modified base **εA**20. (**b**) Insertion of the T7–Hg(II)_2_–**εA**20 base pair into the parallel-stranded DNA duplex. Only the DNA and the Na^+^ counter-ions are displayed, while water molecules are not shown.

Replacing the nucleobases of the **T**:**A** base pair in the centre of the DNA duplex by the modified base pair **T**:**εA** within the geometry from Figure 4 led to the desired initial structure **5′-(^i^GA^i^G^i^G^i^GATA^i^GAAA^i^G)-3′/5-d’(CTCCCTεATCTTTC)-3′**, which is depicted in Figure 6 along with the nucleobase numbering scheme.

The topology of the DNA for classical MD was generated as described in Section 2.2.

### 3.2. Zipping up the DNA by a Dynamic Distance Constraint

**Figure 7:**
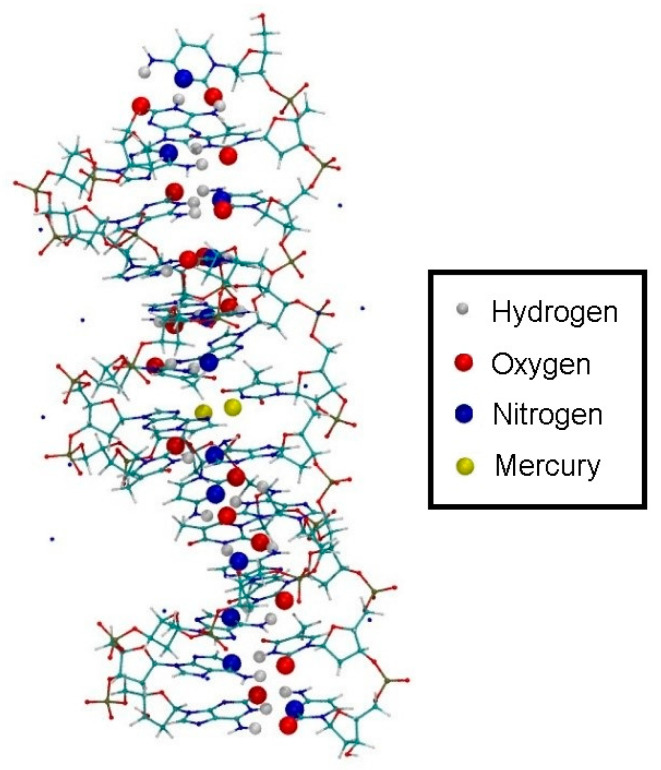
Zipping up the DNA by a dynamic distance constraint across all hydrogen bonds of the non-modified bases. The oxygen, nitrogen and hydrogen atoms shown as large spheres are the constrained partners, where the sum of all bonds represents the dynamic distance.

The initially prepared structure was still in a very high-energy, non-equilibrated state. To relax the geometry, classical MD runs and geometry optimizations were performed using the dynamic distance constraint (Section 2.6.1). This enables fixing the overall hydrogen-bond structure of the DNA during equilibration, while allowing individual hydrogen bond distances to change, which is needed to equilibrate the double helix without it breaking apart into two strands. Because no parametrization of the N–Hg(II) and O–Hg(II) bonds within the modified bases was available, all atoms of the modified base pair (except for the deoxyribose moieties and the phosphate backbone) were fixed at the equilibrium geometry from the optimization from Section 3.1, Figure 4 during purely classical MD, with the computational details given in Section 2.3. With all hydrogen bonds between base pairs selected as a dynamic distance constraint *D*_EQUI_, this dynamic distance was gradually reduced from an initial value of *D*_EQUI_ = 1.88 Å to *D*_EQUI_ = 1.56 Å during an NPT MD simulation with the settings given in Section 2.3 over 10,000 steps, thus “zipping up” the DNA hydrogen bond structure (Figure 7).

### 3.3. Relaxing the DNA Backbone by Geometry Optimization

Simulated annealing starting from the NPT-equilibrated temperature of 300 K was applied during the subsequent 30,000 steps with an annealing factor of 0.99 to slowly relax the DNA duplex. This was followed by a QM/MM geometry optimization [84,85] using CP2K with all constraints released. The computational details are given in Section 2.5.

### 3.4. Relaxing the DNA Structure at Finite Temperature

#### 3.4.1. Structure **1**

**Figure 8:**
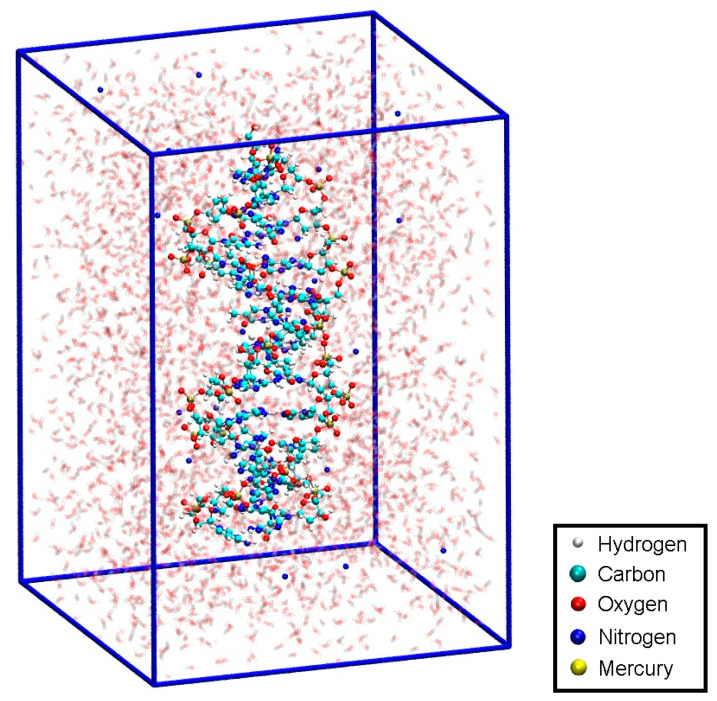
Initial structure **1** within the simulation box, with Na^+^ ions displayed in blue and water molecules shown transparent.

Subsequent to the geometry optimization, a classical MD simulation was performed in the NPT ensemble for pressure equilibration for 1.7 ns and settings from Section 2.3, keeping all atoms of the modified base pair (except for the sugar and phosphate backbone) fixed due to the absence of suitable force field parameters, however releasing the dynamic distance constraint. As the hydrogen bond structure persisted during optimization and MD without the use of the dynamic distance constraint, we considered the DNA duplex to be in a chemical reasonable structure, which we chose as a basis for our further investigations. This resulted in a unit cell of size 43.29 Å × 44.53 Å × 63.33 Å, corresponding to a density of 985 kg/m³. The resulting structure is referred to as structure **1** (Figure 8).

#### 3.4.2. Structure **2**

The dynamic distance method was subsequently employed again as a reaction coordinate to simulate the dissociation of DNA and to determine the corresponding free energy profile. In this case, the dynamic distance *D*_DNA_ comprises all N···H hydrogen bonds of all regular base pairs together with the two N···O distances bridged by the two Hg ions in the metal-mediated base pair (Figure 1a). Thereby all the A-N1···T-H3 and G-H1···C-N3 hydrogen bonds plus the εA-N7···T-O4 and the εA-N6···T-O2 distances in the modified base pair are subject to the dynamic distance constraint. These are the nonoverlapping pairs (NOP) specified in the formula for *D* given in Section 2.6.1.

**Figure 9:**
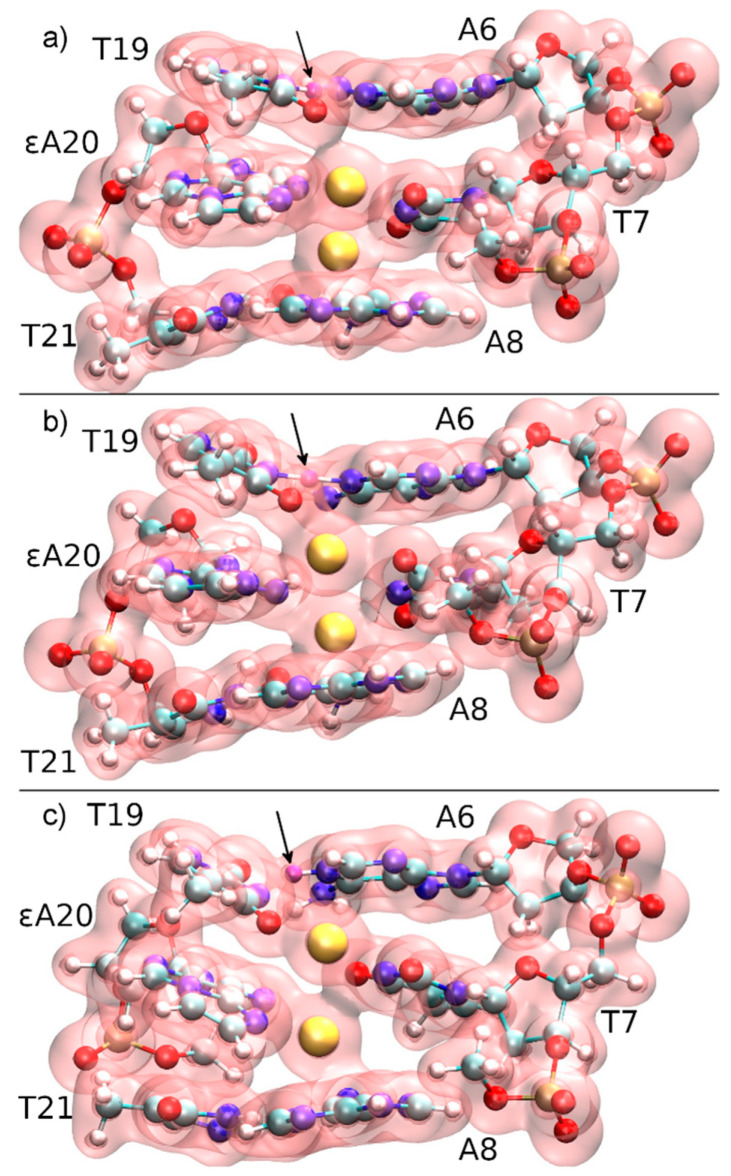
Total electron density of the three base pairs forming the quantum region, together with three phosphate groups neutralizing the subsystem of the constrained QM/MM molecular dynamics starting from structure **1** at *D*_DNA_ = 3.7 Å. The N3H proton of **T**19, which is transferred to **A**6-N1, is marked with an arrow and coloured in purple. (**a**) *t* = 0 fs, the mercury ions (yellow) are initially located within the modified base pair, while Hg2 attracts the electron cloud from **T**19, thus favouring deprotonation. (**b**) *t* = 140 fs, proton transfer transition state. (**c**) *t* = 200 fs, the proton has been transferred to **A**6 and a **T**19–Hg(II)–**T**7 base pair is formed.

**Figure 10:**
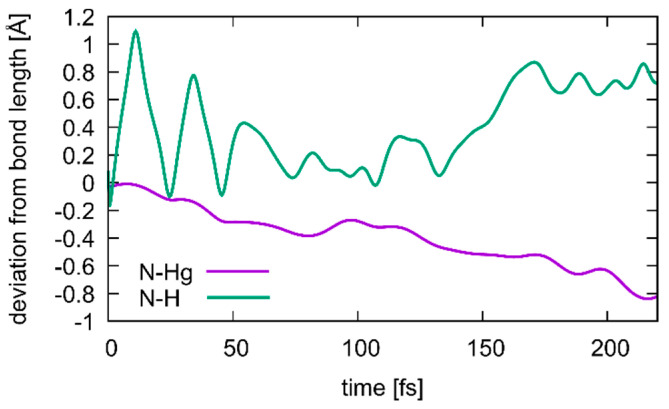
Changes in the N3···H (green) and N3···Hg2 (purple) distances during transition from structure **1** to structure **2**, where a deprotonation of **T**19 occurs simultaneous with the formation of a **T**19–Hg(II)–**T**7 base pair. Correlation between the two distances is visible, with the Hg ion closing in as the proton dissociates, until at 175 ps the **T**19 residue is fully deprotonated.

This collective variable has the advantage of not biasing the dissociation process, i.e., not favouring a particular pathway. At the initial point of *D***_DNA_** = 3.7 Å, a QM/MM molecular dynamics simulation was performed for 4000 steps, resulting in a different structure with Hg1 positioned in-between εA20, **T**7 and **T**8, and a proton being transferred from **T**19 to **A**6 (Figure 9b). Hg2 then cross-links the bases **T**7 and **T**19 originally involved in neighbouring base pairs (Figure 9c). The time evolution of the N3···H and N3···Hg2 distances during deprotonation of **T**19-N3 are depicted in Figure 10, showing that as the proton leaves, its binding position approaches the Hg2 ion. The resulting structure is used as the initial structure **2** for the second dissociation path of the DNA.

#### 3.4.3. Structure **3**

For comparison, an analogous DNA structure without any Hg ions (termed initial structure **3**) was generated by deleting the Hg ions from initial structure **1**, protonating the central thymine residue **T**7 (Figure 1b) and neutralizing the system by the addition of three counter ions. Then a geometry optimization was performed, followed by 500 ps of NPT-MD with settings given in Section 2.3. The resulting structure **3** is shown in Figure S8.

### 3.5. Ab Initio Simulations of Isolated Base Pairs in Solution

#### 3.5.1. Structure **4**

**Figure 11:**
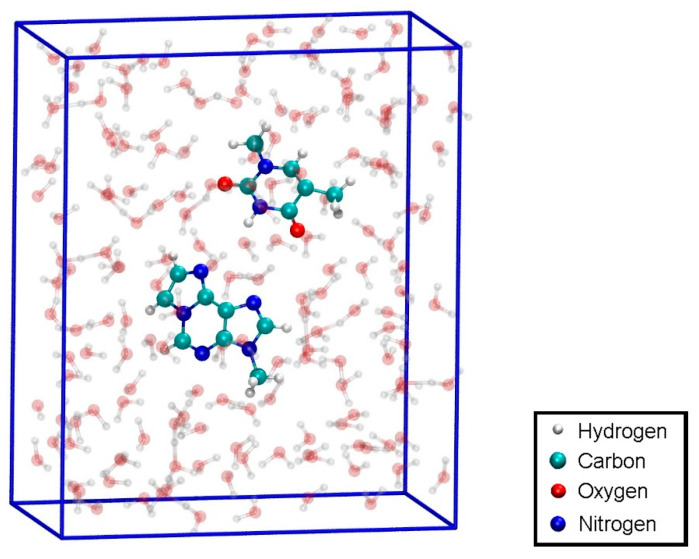
Configuration for the QM dissociation MD of the isolated base pair **T**:**εA** (structure **4**) with the MM-NPT equilibrated water box.

To have access to an all-QM calculation, the isolated modified base pair without Hg (Figure 1b) was solvated by 168 water molecules (Figure 11). The computational details are given in Section 2.3. The force field allowed for a 150 ps NPT equilibration simulation of the solvated base pair. This resulted in a unit cell of size 11.2 Å × 24.2 Å × 20.4 Å, corresponding to a density of 1037 kg/m^3^. This geometry will be referred to as structure **4**.

Structure **4** was used as the initial geometry for an all-QM dissociation simulation of the isolated base pair without Hg ions in-between the bases.

#### 3.5.2. Structure **5**

Adding two Hg ions in-between the bases while removing the proton from T-N3 in structure **4** led to the initial structure for the dissociation simulation of the isolated mercurated base pair (structure **5**).

### 3.6. Free Energy Simulations

Having generated these five different model systems, free energy simulations were performed to analyze the dissociation path and thus, using the principle of micro-reversibility, the formation path of the DNA duplex. QM/MM MD was performed with the settings outlined in Section 2.5 and the dynamic distance as defined in Section 2.6.1.

## 4. Discussion

### 4.1. DNA Dissociation

**Figure 12:**
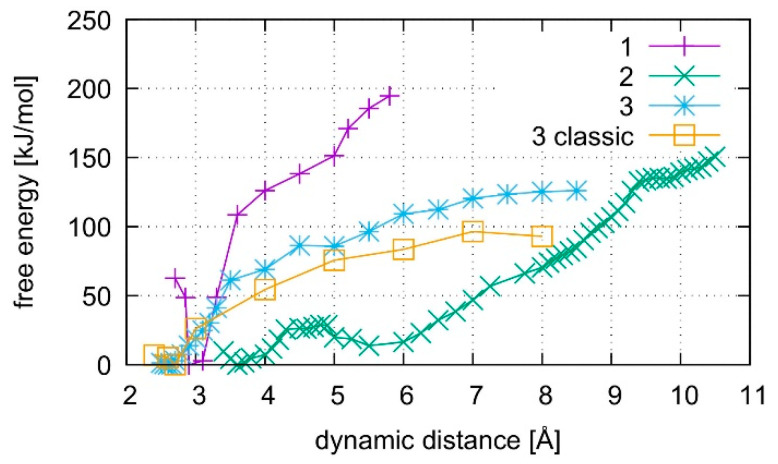
Comparison of free energies for the dissociation of structures **1**–**3**. Structures **1** and **2** contain the dinuclear Hg(II)-mediated base pair, whereas structure **3** is the analogous mercury-free duplex. Structure **1** starts from a QMMM-optimized geometry, whereas structure **2** starts after QMMM-MD, and structure **3** is simulated by both classical and ab initio MD.

**Figure 13:**
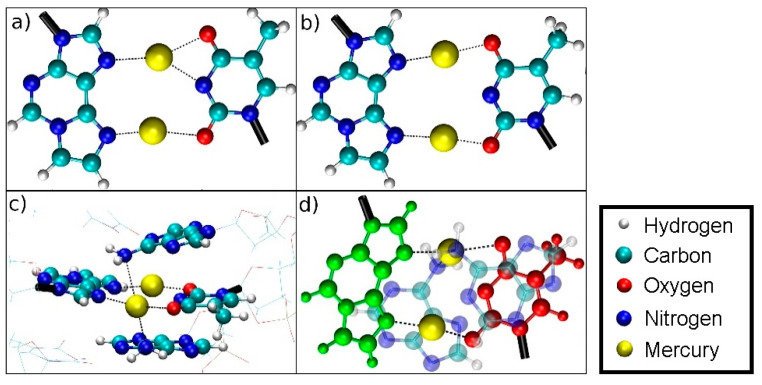
Oscillating bonding patterns of the metal-mediated base pair during the QM/MM MD of structure **2** at *D*_DNA_ = 3.8 Å. Only the base pairs of interest are shown, with bonds to the backbone drawn in black. Water molecules are not displayed. (**a**) NN bonding pattern; (**b**) NO bonding pattern; (**c**) influence of the amino groups of adenine moieties **A**6 and **A**8 on Hg2; (**d**) view of situation c when rotated by 90° with **T**7 drawn in red, **εA**20 drawn in green with the NH_2_ groups of the neighbouring adenine **A**6 and **A**8 residues located on top and below Hg2.

**Figure 14:**
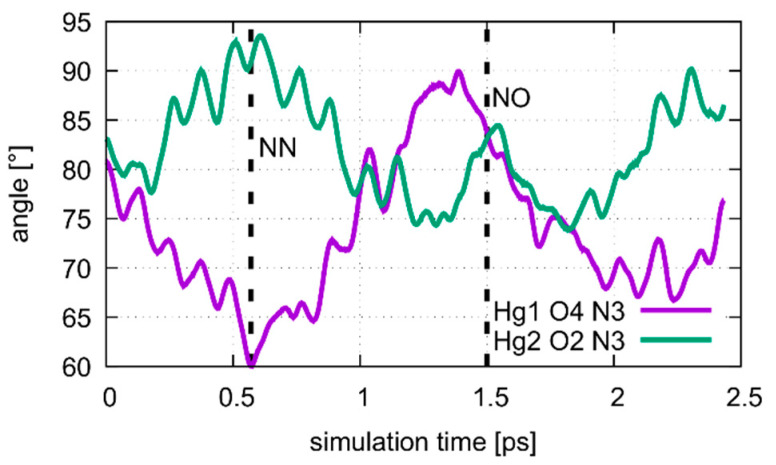
Dynamics of the Hg1···**T**7O4···**T**7N3 and Hg2···**T**7O2···**T**7N3 angles during the oscillation of patterns of the metal-mediated base pair during the QM/MM MD of structure **2** at *D*_DNA_ = 3.8 Å. The **NN** pattern can be identified after 0.6 ps, corresponding to the situation in Figure 13a. At 1.5 ps the system is in the **NO** pattern, depicted in Figure 13b.

The calculated free energy profiles for DNA dissociation are presented in Figure 12. At the lowest energy dynamic distance of *D*_DNA_ = 2.9 Å of structure **1**, the average distance between the carbon atoms C1′ of the deoxyribose attached to the nucleobases of the modified base pair is 10.4 Å. This is somewhat smaller than the experimental value of 11.4 Å reported for a parallel-stranded duplex composed of **A**:**T** base pair in the reversed Watson–Crick geometry [86]. Within structure **2**, where **T**19 was deprotonated and the Hg(II) ions were bound across different base layers, the free energy minimum is found at *D*_DNA_ = 3.6 Å with an average C1′···C1′ distance of 11.8 Å, which is closer to the experimental value. During the simulation of structure **2**, both the **NO** and the **NN** bonding pattern are observed, with the system oscillating between the two minima (Figure 13 and Figure 14). The first free energy minimum observed around *D*_DNA_ = 3.6 Å arises because the amino groups of adenine residues **A**6-N6H_2_ and **A**8-N6H_2_ involved in one of the base pairs adjacent to the metal-mediated one stabilize the Hg2 ion in-between them.

In all simulations, the modified base pair proved to be the weak spot of the DNA duplex, opening before the other base pairs. The bonding was found to be weakest if no mercury was incorporated into the DNA as can be seen from the free energy curves in Figure 12. As the dissociation simulation of structure **3** led to a breaking of the inner-pair hydrogen bonds of the artificial base pair (see Figure S3), the artificial base pair in case of structure **3** can be considered dissociated. For structures **1** and **2**, only a change of bonding patterns occurred; thus, the DNA with Hg(II) can be considered more stable. To check for a possible influence of the classical force field parameters, the dissociation MD of structure **3** was repeated by a purely classical force-field approach. This was possible because no mercury ions are present in structure **3.** Ten points along the trajectory of structure **3** were sampled for 55 ps, each, by classical MD.

Structure **1,** where the calculations started from the optimized QM subsystem, emerges as the most stable structure, exhibiting the biggest energy gradient and thus the largest required force to promote the system out of its initial bonding pattern. It is followed by structure **2**, which exhibits a second energy minimum along the dissociation path at *D*_DNA_ = 5.5 Å. At this point, Hg1 is still located in the modified base pair, whereas Hg2 forms a diagonal **T**7–Hg(II)–**T**19 base pair involving nucleobases that were originally located in neighbouring base pairs. Such an unusual metal-mediated base pair was recently observed in a crystal structure, too [87]. This bonding pattern was enabled by deprotonation of **T**19, which is favoured by the presence of Hg(II) in the adjacent modified base pair. Structure **3** shows the weakest binding in the classical description. Its QM description led to lower binding energies than observed in the metal-mediated DNA duplexes. At *D*_DNA_= 8.5 Å, structure **3** opened a cavity at the modified base pair, allowing water to interact with the base pairs of the DNA duplex.

All computed free energy differences lie in the range of 100–200 kJ/mol, which are typical binding energies of DNA base pairs [88]. These calculated energies correspond to the dissociation process depicted in Figures S1–S3 for the simulations of the whole DNA and to the binding energy of the isolated base pair in water from Section 4.2. As the simulated partial dissociation in the whole DNA mainly affects the modified base pair, the calculated energies are indicative of the amount of energy required to induce the observed change of bonding patterns within the modified base pair.

**Figure 15:**
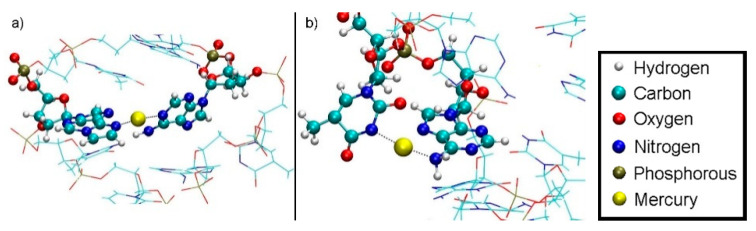
Section of final geometry of structure **1** at *D*_DNA_ = 5.8 Å with no water displayed. (**a**) Hg2 is bound between **A**6-N1 and **εA**20-N6 inside the double helix. (**b**) Hg1 is bound between **T**7-N3 and **A**8-N6 outside the double helix, with the N6 atom of adenine being hybridized in an *sp*^3^-like state to engage in a coordinate bond with Hg1. The base pairs bulge of the DNA helix and are dangling in the water, thus forming a contact of the hydrophobic core to the water environment.

Within structures **1** and **2**, the Hg(II) ions did not leave the DNA during the dissociation simulation, but changed their bonding pattern. However, structure **1** exhibited a dissociation path that allowed Hg2 to get into contact with water. This dissociation path can be analysed as follows. From the initial geometry of being situated within the modified base pair, the final geometries for structure **1** at *D*_DNA_ = 5.8 Å showed Hg2 in an **A**6–Hg(II)–**εA**20 environment (Figure 15a). The median distances from Hg2 to the coordinated nitrogen atoms are 2.15 Å to **εA**20-N6 and 2.17 Å to **A**6-N1. Hg1 is involved in a **T**7–Hg(II)–**A**8 pair dangling on the side of only one strand with a median distance from the coordinated nitrogen atoms to Hg1 of 2.17 Å (**T**7-N3) and 2.12 Å (**A**8-N6) (Figure 15b). These values suggest the formation of coordinate bonds to the Hg ions. The NH_2_ group of **A**8 changes its hybridization from a *sp*^2^-like planar state to a *sp*^3^-like tetrahedral state, with Hg1 acting as the fourth binding partner. Such a hybridization shift of the exocyclic amino group of adenine has been reported before in structurally characterized metal complexes [89].

**Figure 16:**
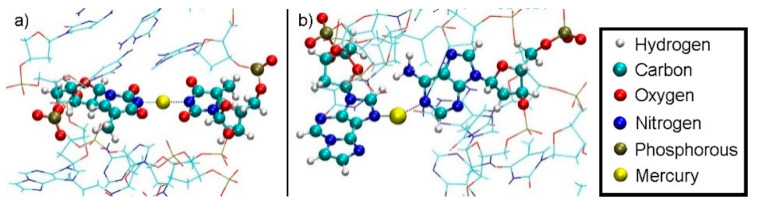
Final geometry of structure **2** at *D*_DNA_ = 10.4 Å with no water displayed. (**a**) Hg2 is bound between **T**19-N3 from the base pair originally adjacent the modified base and **T**7-N3 from the modified base pair. **T**19 transferred its N3–H proton to **A**6-N1 during the equilibration MD due to the presence of Hg(II). (**b**) Final geometry of structure **2** after thermodynamic integration at *D*_DNA_ = 10.4 Å with water not displayed. Hg1 is bound by **A**8-N1 from the base pair originally below the modified base and **εA**20-N7.

The dissociation MD of structure **2** proceeded to a final value of *D*_DNA_ = 10.4 Å, when the quantum-mechanically treated Hg1 came into contact with classically treated water. At this point, Hg2 is involved in a **T**7–Hg(II)–**T**19 pair, with equal Hg–N3 distances of 2.15 Å on either side (Figure 16a). In this structure, Hg1 is contained in an **εA**20–Hg(II)–**A**8 pair with median distances from the nitrogen atoms coordinated to Hg(II) of 2.20 Å (**A**-N1) and 2.18 Å (**εA**-N3), which are close to the distances found in the **εA**20–Hg(II)–**A**6 base pair in structure **1**. All distances suggest coordinate bonding of the Hg(II) ions (Figure 16b).

**Figure 17:**
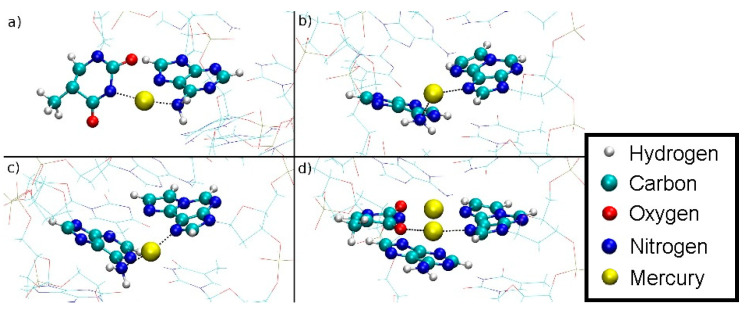
Simulated path of the Hg1 into the DNA duplex for structure **1** as derived from assuming microscopic reversibility of the dissociation of structure **1**. The *sp*^3^-like hybridized amino group of **A**8 can clearly be discerned based on the orientation of its protons. (**a**) *D*_DNA_ = 5.8 Å**,** Hg(II) outside the DNA duplex in water, attached to the NH_2_ group of **A**8 and a dangling **T**7. (**b**) *D*_DNA_ = 4.5 Å, the NH_2_ group is rotated about the C6–N6 bond and the Hg(II) is being attached to **εA**20. (**c**) *D*_DNA_ = 3.6 Å, further rotation of the NH_2_ group. (**d**) *D*_DNA_ = 3.1 Å, inclusion of Hg1 into the metal-mediated base pair with Hg2 also shown. The NH_2_ group is now *sp*^2^-hybridized.

The only migration of an Hg ion from the DNA inner core to the water solvent occurred in structure **1**. The inverse of the pathway out of the DNA duplex can be considered a possible pathway into the duplex, using the principle of microscopic reversibility. The transition out of the DNA duplex is mediated by the exocyclic amino group of **A**8-N6H_2_ from the layer below the metal-mediated base pair. Hg1 approaches the NH_2_ group, thus leading to an *sp^3^*-like hybridization of its nitrogen atom. Being attached to this moiety, which can rotate due to its single bond to the adenine, Hg1 can rotate out of the DNA core, while remaining attached to this group (Figure 17).

The distance from Hg1 to **A**8-N6 is shown in Figure S9. During the rotation, the distance oscillates around at a mean value of 2.2 Å, while after the rotation, the Hg ion is coordinated by **T**7-O4 and **εA**20-N7 and the distance from Hg1 to **A**8-N6 reaches 2.8 Å, suggesting that the bond to the NH_2_ group is broken.

It is tempting to speculate that the relative location of the neighbouring adenine residue with respect to the **εA**:**T** mispair is important for the incorporation of the second Hg(II) ion into the dinuclear base pair. This would be in good agreement with the observation that only a mononuclear Hg(II)-mediated base pair is formed between **εA** and **T** when the complementary sequences are arranged in an antiparallel-stranded manner, i.e., when the relative orientation of the neighbouring base pairs is different (see Supporting Information for details).

### 4.2. Dissociation of the Isolated Base Pair

**Figure 18:**
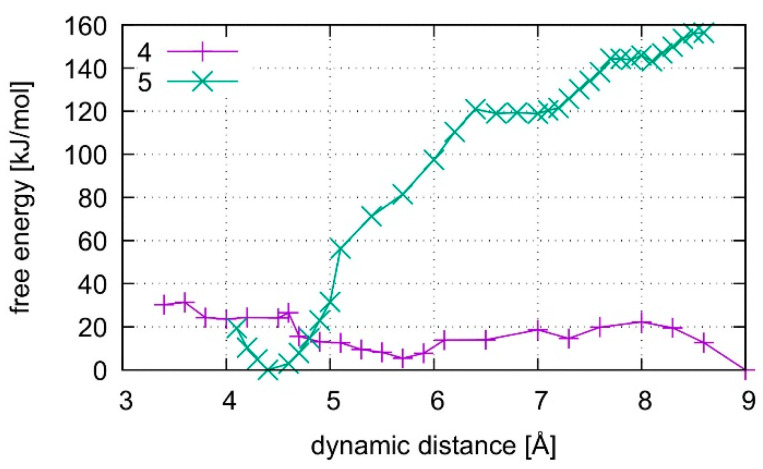
Comparison of the dissociation free energy in liquid water for the isolated **T**:**εA** base pair where base pair **4** does not contain any Hg(II) ions whereas structure **5** is the dinuclear Hg(II)-mediated base pair. The introduction of Hg(II) leads to the formation of a metal-mediated base pair, whereas the Hg(II)-free base pair dissociates without the need of further energy.

**Figure 19:**
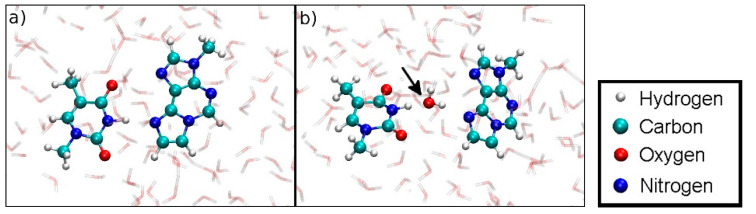
(**a**) Initial geometry at *D*_ISO_ = 3.6 Å of the dissociation MD for the Hg(II)-free hydrogen-bonded base pair (structure **4**). (**b**) Dissociation is favoured due to the involvement of a water molecule in hydrogen bonding, which is not possible within a DNA duplex.

The isolated **εA**–Hg(II)_2_–**T** base pair with Hg(II) ions incorporated (structure **5**) exhibited a binding free energy of about 160 kJ/mol, while the isolated non-metal-modified base pair **εA**:**T** (structure **4**) separated without any additional energy in a water environment (Figure 18). The latter can be explained by the fact that water molecules approaching from above or below have the propensity to form strong hydrogen bonding with the individual bases of the **εA**-:**T** pair, thus favouring dissociation (Figure 19b). Within the DNA helix, this effect is compensated by the hydrophobic inner DNA core, which blocks access of water from above or below, thus leading to a substantial binding energy of about 100 kJ/mol (MM) and 125 kJ/mol (QM/MM) for the non-metal-mediated DNA duplex (Figure 12).

**Figure 20:**
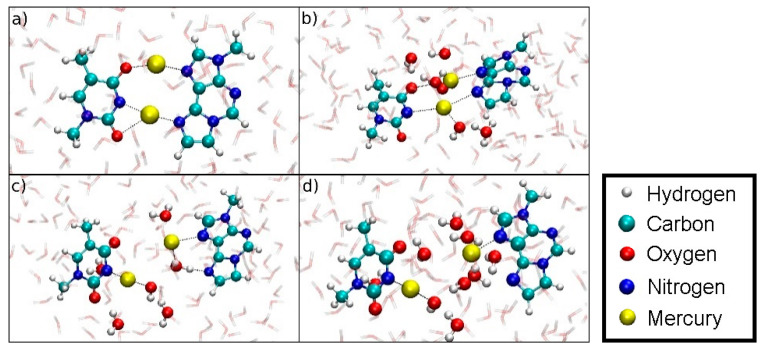
Snapshots of the dissociation MD for the metal-mediated base pair (structure **5**). (**a**) *D*_ISO_ = 4.4 Å, initial geometry. (**b**) *D*_ISO_ = 6.4 Å, formation of an H_3_O^+^ and an OH^–^ close to Hg2. (**c**) *D*_ISO_ = 7.3 Å, coordination of a water molecule at the former **εA**-N6 binding site of Hg2, while the OH^–^ coordinates the **T**-N3-bonded Hg1. (**d**) *D*_ISO_ = 8.6 Å, final geometry of the dissociation MD. Hg1 remains attached to **T**-N3 and carries an additional OH^–^ ligand, Hg2 binds **εA**-N7, while the water molecules form a bridge with their oxygen atoms pointing towards to the positively charged Hg(II) and their protons facing the electronegative O and N atoms of the nucleobases.

Along the dissociation path of structure **5,** the dinuclear base pair splits up such that each base keeps an Hg(II) ion attached to it. Hg1 bonded to **T**-N3 coordinates an additional OH^−^ formed from a nearby water molecule. The former binding site of Hg1 at **εA** is filled up by a hydrogen-bonded water molecule, pointing its oxygen atom towards Hg2. At *D*_ISO_ = 8.6 Å, the attractive Coulomb interactions between the bases are screened by water molecules, facilitating further dissociation (Figure 20).

## 5. Conclusions

Free energy QM and QMMM molecular dynamics simulations have been performed of an experimentally established parallel-stranded DNA duplex bearing a **T**–Hg(II)_2_–**εA** base pair. Two main bonding patterns have been identified for the mercury(II)-mediated base pair. For the most stable structure **2**, the C1′···C1′ distance of the carbon atoms involved in the glycosidic bonds is close to the experimental value derived from a related experimental structure [86]. While the isolated nucleobases **T** and **εA** do not form a base pair in the absence of Hg(II), the inclusion of Hg(II) leads to a stable dinuclear metal-mediated base pair. If neighbouring base pairs are considered in the calculations, the amino groups of their adenine nucleobases influence the bonding pattern by binding to the Hg(II) from above and below. This unexpected structural insight shows that the precise location of a metal ion within a metal-mediated base pair is not necessarily identical to that in the ideal geometry of the isolated base pair but may also depend on the identity of the adjacent canonical base pairs.

In the complete DNA duplex, the artificial base pair proved to be the weak point in both classical and quantum mechanics simulations if no Hg(II) is incorporated. Upon to the incorporation of Hg(II), the base pair is strengthened. In the dissociation simulations of the Hg(II)-bound DNA duplex, a shift in bonding patterns is observed, with the Hg(II) ions engaging in the original **T**–Hg(II)_2_–**εA** base pair, in an inter-planar **T**–Hg(II)–**T** base pair, or in an unprecedented **A**–Hg(II)–**εA** base pair. Irrespective of the precise coordination environment of the Hg(II) ions, the Hg(II)-containing DNA is predicted to be more stable than its non-metalated counterpart.

During one simulation, a mercury ion was transferred from the inner DNA region to the surrounding water, where the amino group of an adjacent adenine moiety acted as a turnstile to transport the mercury(II) ion out of the DNA. Due to the principle of microscopic reversibility, this adenine residue can be postulated to act as a gateway for Hg(II) during the formation of the metal-mediated base pair. This indicates another important contribution of the oligonucleotide sequence on the mechanism of metal-mediated base pair formation, which will need to be investigated in detail in future experiments. This is in good agreement with the experimental observation that in a different sequence context, only one Hg(II) is incorporated into the **εA**:**T** pair. The present results may thus prove useful in guiding the future design of Hg-modified DNA.

As Hg(II) has a linear coordination environment, the findings of this paper concerning the structural arrangement can only be transferred to metal ions which coordinate linearly, which is not the case for the other metal ions mentioned in the introduction, except for Ag(I). Similarly, they are not applicable to Ag(I), because as a monovalent cation it is less capable of deprotonating aqua ligands to hydroxido ligands. However, the observed turnstile mechanism might be relevant to the incorporation of other metal cations into DNA, as they can bind to the lone electron pair of the adenine amino group.

Aside from the chemical insights gained, it is worth noting that the dynamic distance constraint proved to be a useful method to investigate bonding patterns in systems with competing (hydrogen) bonds—not only for quantitative free energy simulations, but also for constructing DNA geometries with predefined hydrogen bonding patterns. Since this is a common issue in the investigation of solvated biomolecules, the current approach should also be helpful for many other systems.

## Supplementary Materials

The following are available online, Figure S1: Final geometry of structure 1, Figure S2: Final geometry of structure 2, Figure S3: Final geometry of structure 3, Figure S4: Lagrange multipliers for path 1, Figure S5: Lagrange multipliers for path 2, Figure S6: Lagrange multipliers for path 3, Figure S7: Energies and end-to-end distances of optimized geometries for different functionals, Figure S8: DNA structure without Hg, Figure S9: A8-N6···Hg(II) distance, PDB structure of the DNA, topology of the DNA for use in CP2K, Cartesian coordinates corresponding to Figure 4 and Figure 5.
