# Supplementary material for: Dynamic Structure and Stability of DNA Duplexes Bearing a Dinuclear Hg(II)-Mediated Base Pair
Source: Molecules. 2020 Oct 26;25(21):4942. doi: 10.3390/molecules25214942 (PMC7663159; doi:10.3390/molecules25214942)
Supplement: Supplementary file 1 [file molecules-25-04942-s001.pdf]

SUPPORTING INFORMATION

Jim Bachmann,[a] Isabell Schönrath,[b] Jens Müller*[b], Nikos L. Doltsinis*[a]

[1] Westfälische Wilhelms Universität Münster, Institut für Festkörpertheorie and Center for Multiscale Theory and Computation, Wilhelm-Klemm-Straße 10, 48149 Münster, Germany.

[2] Westfälische Wilhelms Universität Münster, Institut für Anorganische und Analytische Chemie, Corrensstr. 28/30, 48149 Münster, Germany.

Table of Contents

Fig. S1-S3: Final geometries of the dynamic distance constraint simulations………….. 3
Fig. S4-S6: Running average of the Lagrange multipliers ………………………… 6
Fig. S7: Functional comparison for optimization ……………………………………… 10
Fig. S8: DNA structure without Hg …………………………………………………….. 10
Fig. S9: A8-N6···Hg(II) distance …………………………………………………….. 10

Experimental Observation …… ……………………………………………… 11

Fig. S10: Melting curves, melting temperatures, CD spectra …………………………… 12

Materials and Methods …………………………………………………………… ………… 13

Fig. S11: MALDI-TOF mass spectrum of 5'-d(GAA AGA TAG GGA G)-3' ………… 13
Fig. S12: MALDI-TOF mass spectrum of 5'-d(CTC CCT εATC TTT C)-3' ……………. 14

References ……………………………………………………………………………… 14

Files: Base pair coordinates corresponding to Fig. 4 and 5,
DNA geometry and topology for CP2K ……………………………………… 14

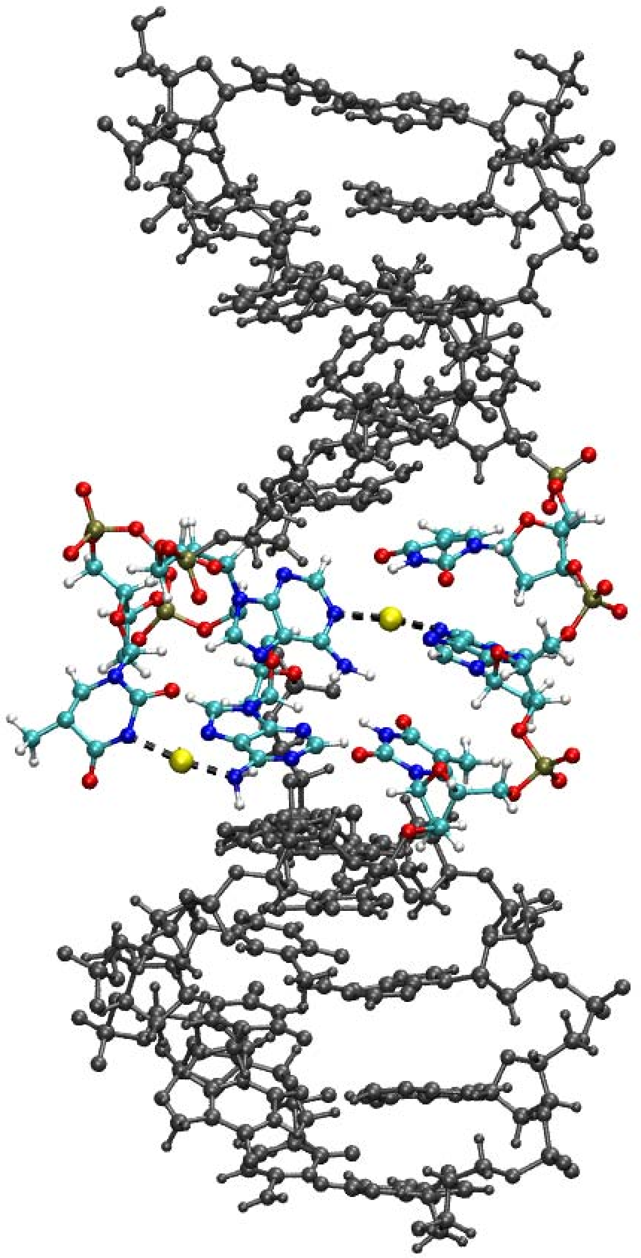

Figure S1. Final geometry of structure **1** after thermodynamic integration at $\mathbf{D}_{\mathrm{DNA}}$ = 5.8 Å with water not displayed. The bond structure of the modified base pair has changed, with Hg1 in between **A**8-N6 and **T**7-N3 from the modified base, where **T**7 is dangling in the water outside of the DNA core in a **T7-Hg(II)-A**8 bond (lower Hg in the figure). The Hg2 is bound inside the DNA duplex in a **A6-Hg(II)-εA**20 bond attaching to **A**6-N1 and **εA**20-N6 (upper Hg in the figure).

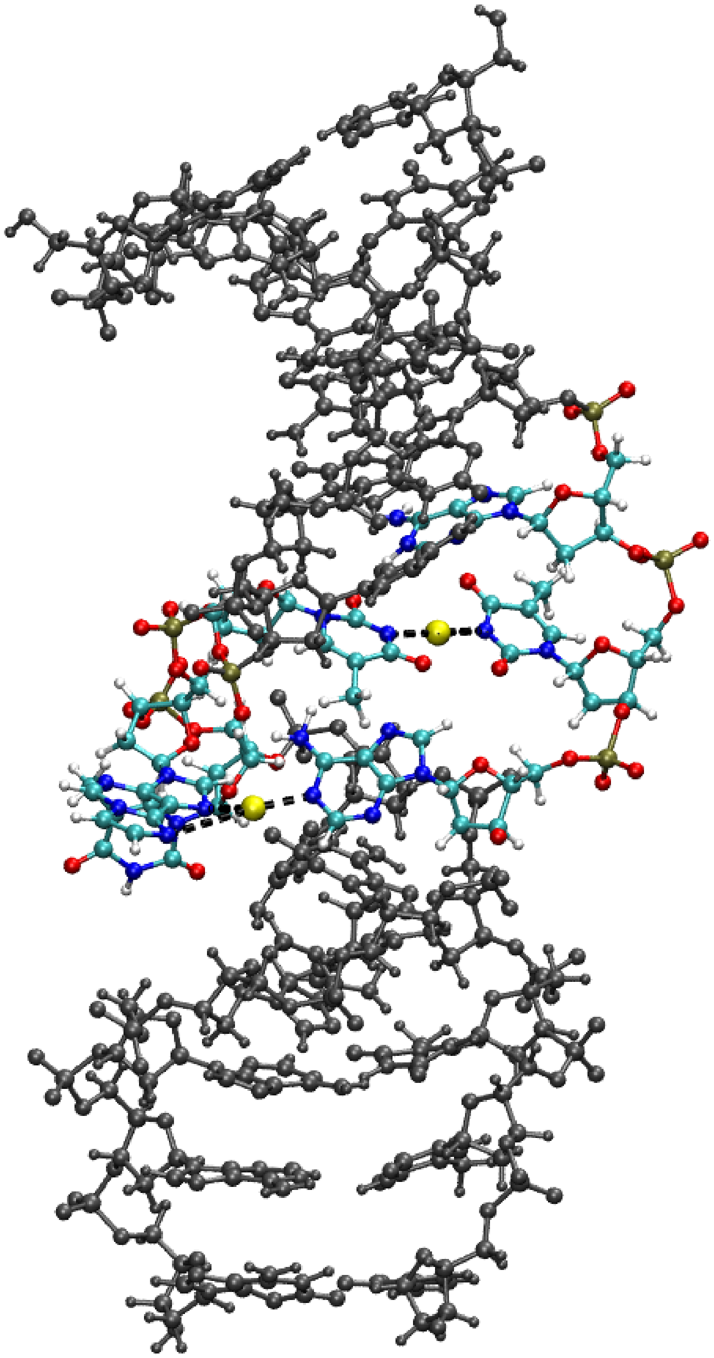

Figure S2. Final geometry of structure **2** after thermodynamic integration at $\mathbf{D}_{\mathrm{DNA}}$ = 10.4 Å with water not displayed. The bond structure of the modified base pair has changed, with Hg1 in between **εA**20-N7 and **A**8-N1 of the base layer under the modified base pair (lower Hg in the figure). Hg2 is bound forming a **T7-Hg(II)-T**19 bond inside the DNA duplex (upper Hg in the figure) with the Hg2 attaching to **T**7-N3 and **T**19-N3.

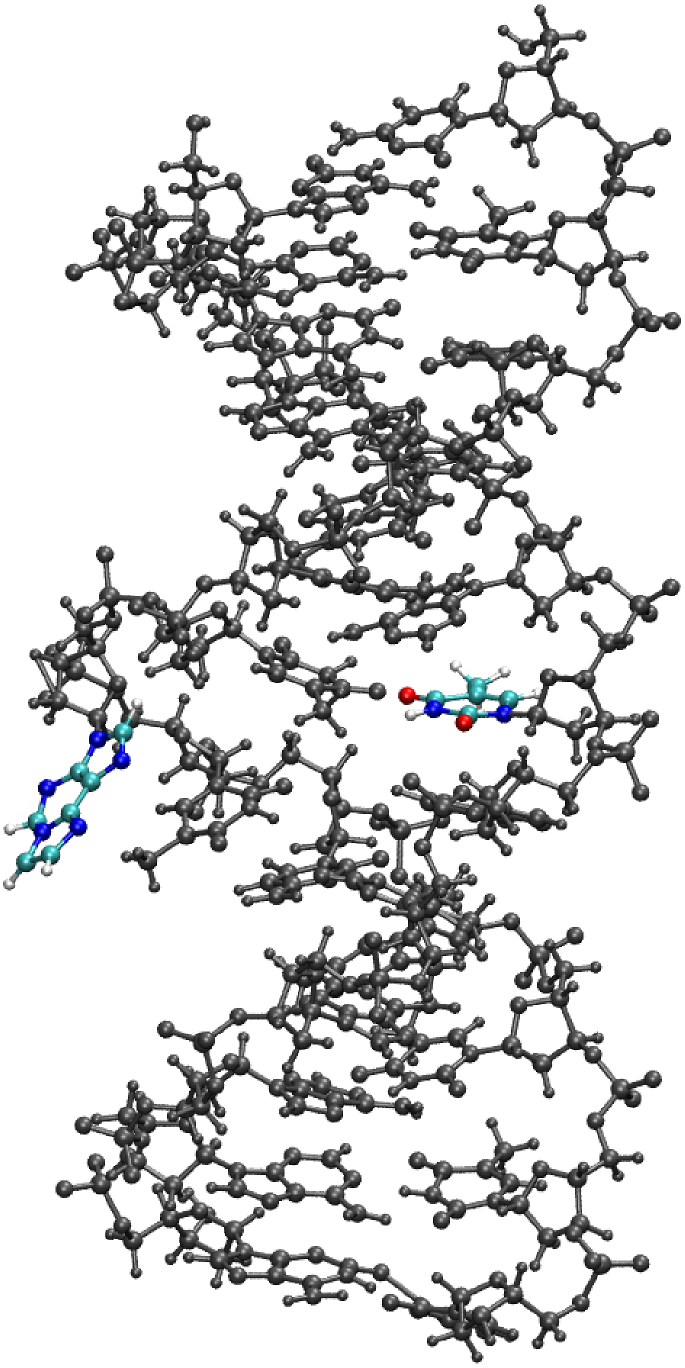

Figure S3. Final geometry of structure **3** after thermodynamic integration at D_{DNA} = 8.5 Å with water not displayed. A cavity has opened at the modified base pair with **εA** dangling into the surrounding water.

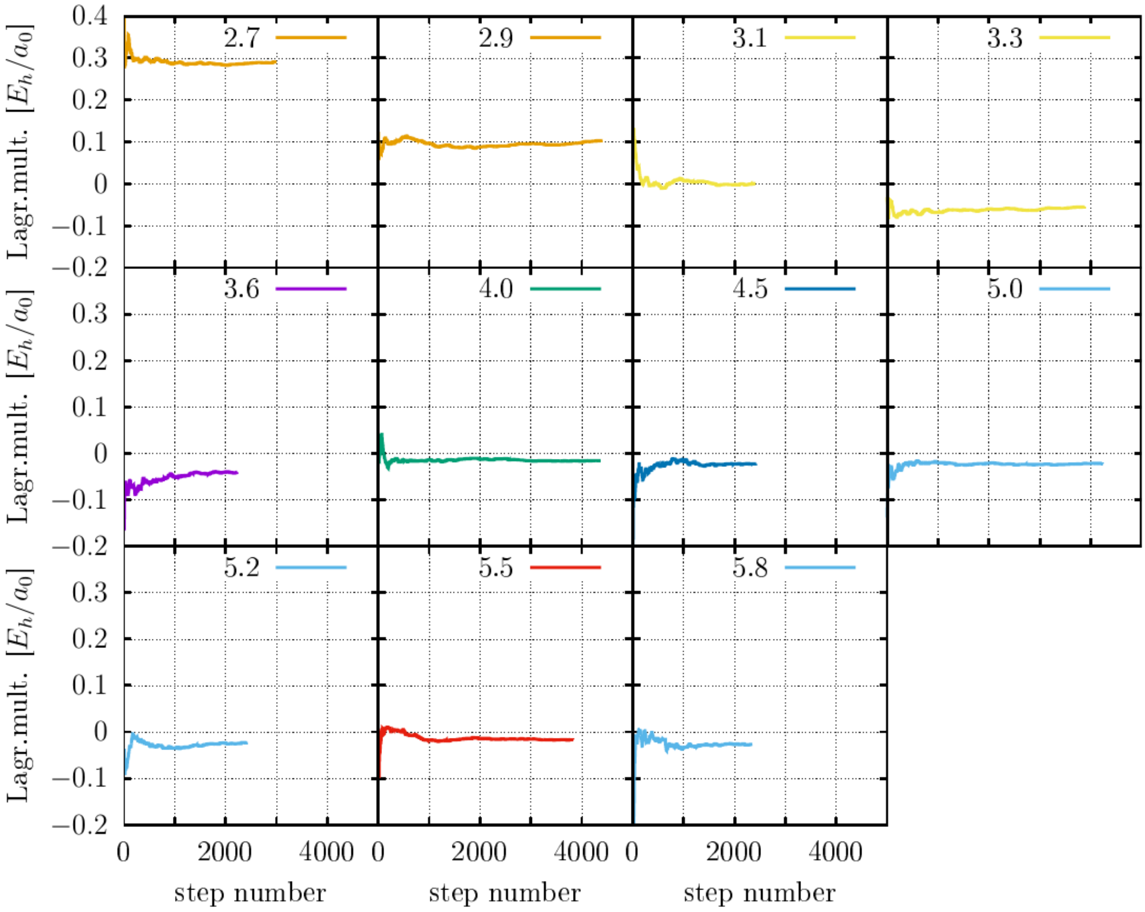

Figure S4. Running average of the Lagrange multiplier in E_H/a_0 (Hartree energy E_H per Bohr radius a_0) versus step number for path 1 at each constraint value.

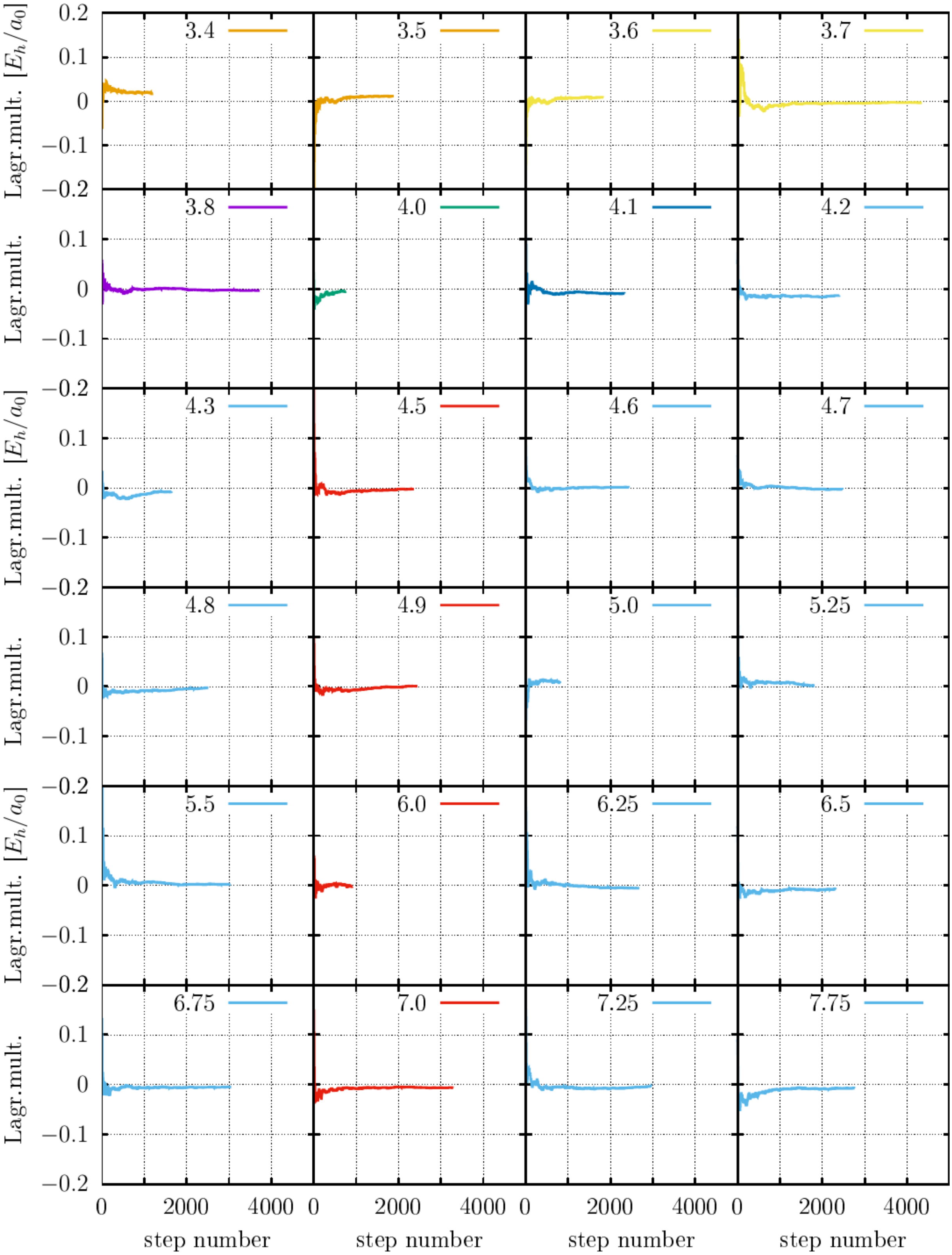

Figure S5. Running average of the Lagrange multiplier in E_H/a_0 (Hartree energy E_H per Bohr radius a_0) versus step number for path 2 at each constraint value.

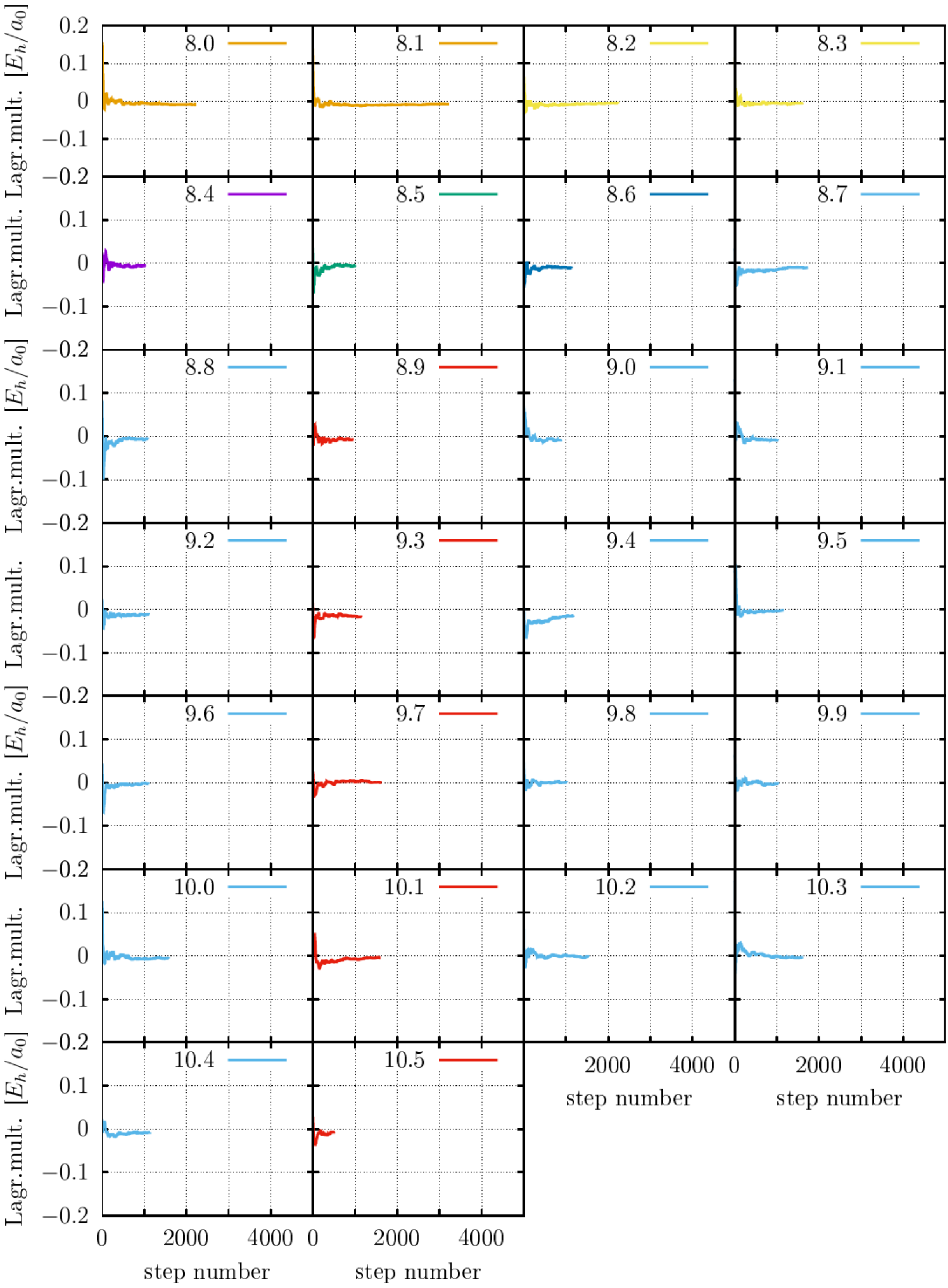

Figure S5. continuation: Running average of the Lagrange multiplier in E_H/a_0 (Hartree energy E_H per Bohr radius a_0) versus step number for path 2 at each constraint value.

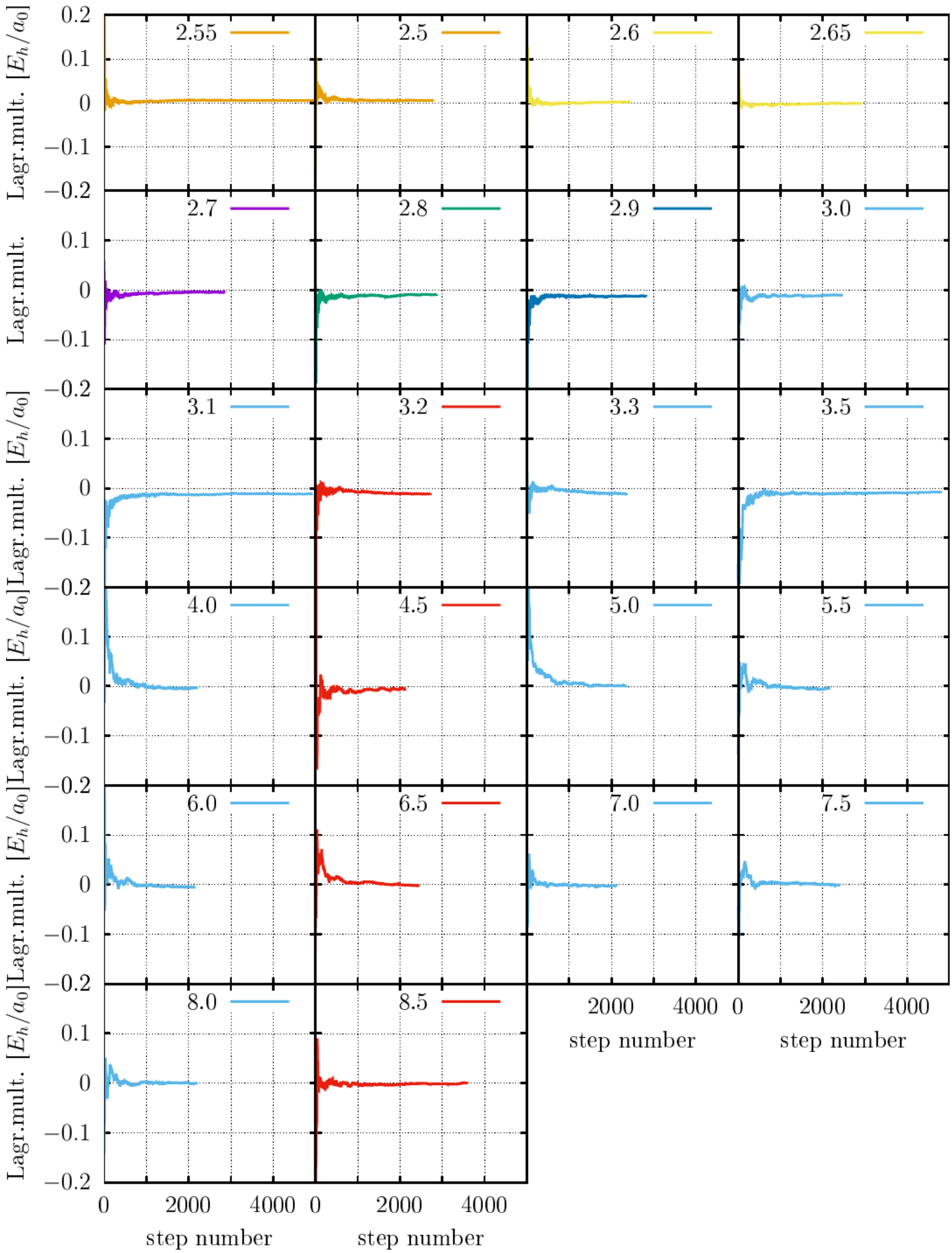

Figure S6. Running average of the Lagrange multiplier in E_H/a_0 (Hartree energy E_H per Bohr radius a_0) versus step number for path 3 at each constraint value.

	PBE0-TZVP-ZORA	BP86-TZVP-ZORA	BLYP-SDD	B3LYP-SDD	BP86-SDD	PBE-SDD	PBE0-SDD	PBE0-SDDALL	PBE0-TZVP	PM6
Energy NN (eV)		-1135682,4	-37583,78	-37595,66	-37602,42	-37561,70	-37559,49	-13476,34	-37575,31	35,32
Distance R R' NN (Å)		12,23	12,23	12,23	12,23	12,23	12,23	12,23	12,23	12,23
Energy NO (eV)	-1135527,88	-1135684,5	-37584,31	-37595,91	-37602,72	-37561,97	-37559,64	-13476,51	-37575,89	32,42
Distance R R' NO (Å)	11,55	11,67	11,98	11,85	11,89	11,87	11,75	11,69	11,55	12,52
dE (eV)		-2,17	-0,53	-0,24	-0,31	-0,27	-0,15	-0,17	-0,57	-2,90
Opt finds global Min.	yes	yes	yes	yes	no	no	no	no	no	no

Figure S7. Summary of the optimizations of the isolated **T-2Hg(II)-εA** base pair with a PCM continuum solvation model. BLYP and B3LYP find the global minimum without the need for ZORA corrections, where the R R' distance is close to the one obtained with B86-ZORA. dE labels the energy difference between the NN and the NO bonding pattern, where the NN pattern is identified as the global minimum for the isolated base pair.

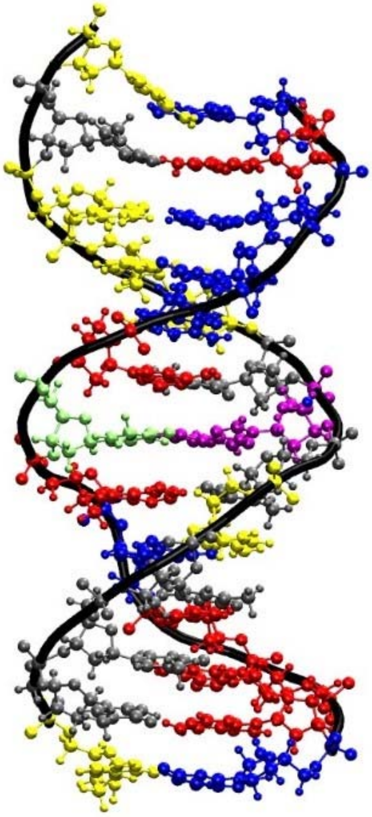

Figure S8. Initial structure 3 for the dissociation MD without Hg in the modified base pair structure with Guanine blue, Adenine red, Thymine grey and Cytosine yellow. Thymine within the modified base pair is drawn in lime and εA in purple. All inner hydrogen bonds between DNA bases are realized as expected for an equilibrium structure.

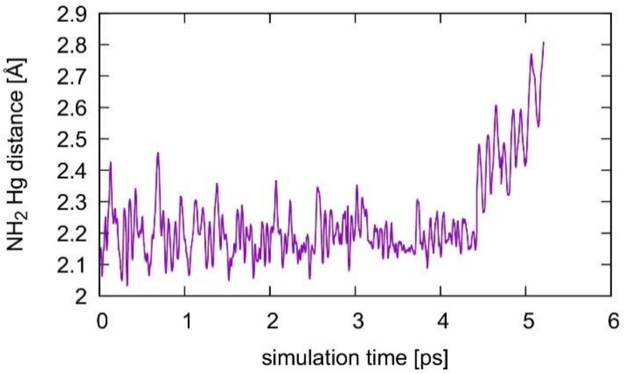

Figure S9. A8-N6···Hg(II) distance during the formation of structure **1** as derived from assuming microscopic reversibility of the dissociation of structure **1** (shown here from D_{DNA} = 5.8 Å to D_{DNA} = 3.1 Å).

Experimental observation of the formation of a mononuclear T–Hg(II)–εA base pair

The previously investigated DNA duplex 5′-d(^{i}GA^{i}G ^{i}G^{i}GA **T**A^{i}G AAA ^{i}G)-3′ / 5′-d(CTC CCT **εA**TC TTT C)-3′ contains base pairs composed of isoguanine (^{i}G) and cytosine to stabilize the desired parallel-stranded orientation of the complementary strands.[1] As shown in this manuscript, one of the adenine residues directly adjacent to the central **T**:**εA** base pair seems to play an important role in the mechanism of incorporation of the second Hg(II) ion. We were therefore interested in probing this hypothesis by investigating a highly similar sequence that nevertheless contains a different local environment of the **T**:**εA** mispair. As a result, the respective antiparallel-stranded duplex (with isoguanine being replaced by guanine) 5′-d(GAG GGA **T**AG AAA G)-3′ / 3′-d(CTC CCT **εA**TC TTT C)-5′ was synthesized and investigated. Even though the exact structure of this duplex was not calculated, it is well established that parallel- and antiparallel-stranded duplexes differ in their structures.[2]

In analogy to our previous investigations of the parallel-stranded duplex,[1] the antiparallel-stranded duplex was investigated at pH 6.8 and at pH 9.0. Figure S10 shows the melting curves and melting temperatures T_m of this antiparallel-stranded duplex depending on the amount of Hg(II) present in solution at these two pH values. Under both conditions, biphasic melting is observed in some of the experiments, particularly at the onset of the experiments (Figure S10a). As a result, the melting temperatures T_m cannot always be determined with high precision. Nonetheless, the data convincingly show that only the addition of the first equivalent of Hg(II) leads to a significant increase in duplex stability (Figure S10b), with excess Hg(II) either having a minor additional stabilizing (pH 6.8) or even destabilizing (pH 9.0) effect. This is a strong indication of the formation of a mononuclear **T**–Hg(II)–**εA** base pair and rules out the incorporation of a second Hg(II). The CD spectra of the duplex clearly show that the regular B-DNA topology is adopted irrespective of the absence or presence of Hg(II) (Figure S10c).[3] Hence, it can be concluded that while a parallel-stranded duplex gives rise to the formation of a dinuclear Hg(II)-mediated base pair, an antiparallel-stranded duplex of essentially the same composition gives a mononuclear Hg(II)-mediated base pair only. This observation is in good agreement with the computationally predicted involvement of a neighbouring adenine residue in the incorporation of the second Hg(II) ion.

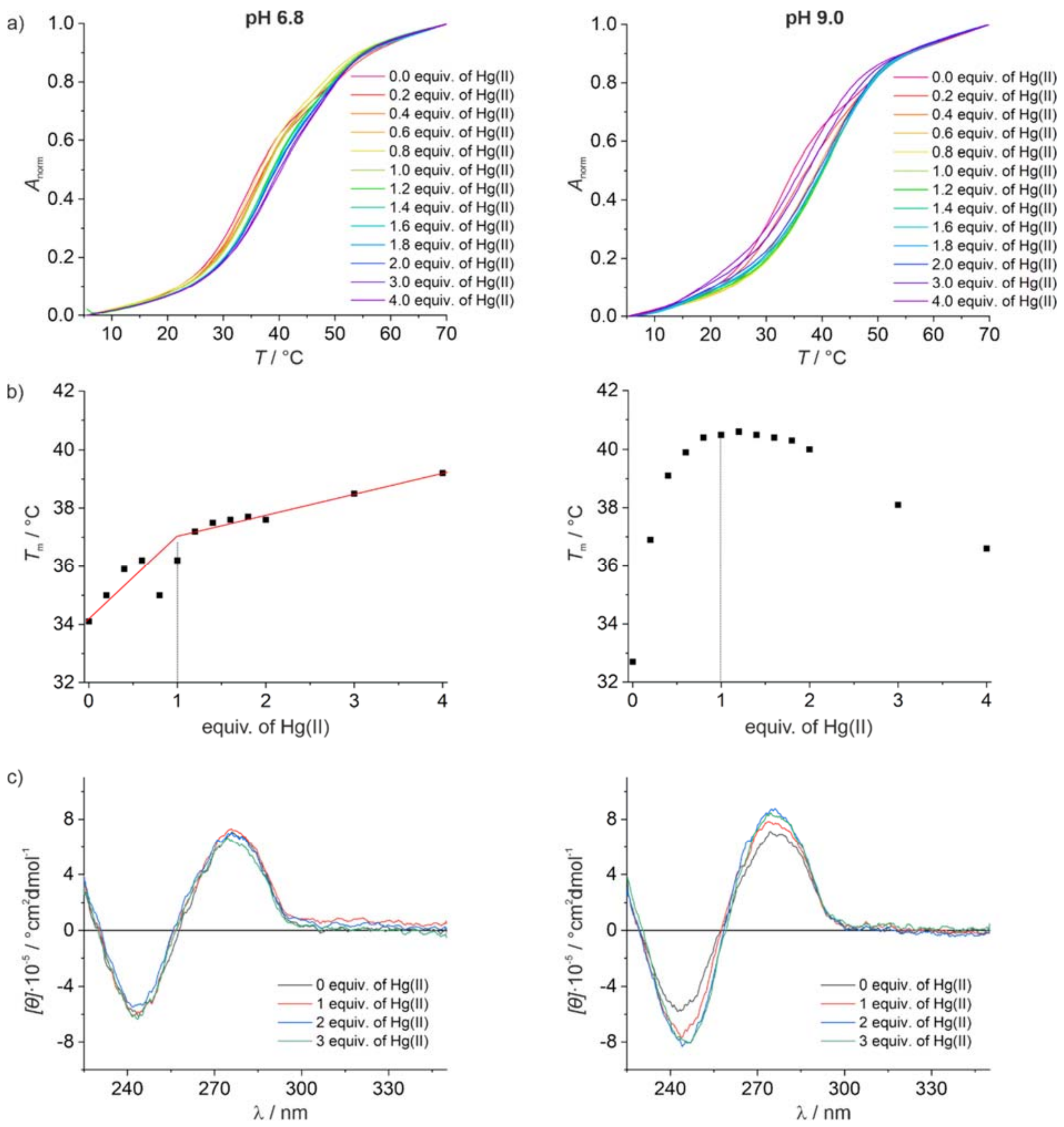

Figure S10. a) Melting curves, b) melting temperatures, and c) CD spectra of the antiparallel-stranded duplex at increasing amounts of Hg(II). The data for pH 6.8 are shown in the left column, those for pH 9.0 in the right column. Experimental conditions: 3 μM DNA duplex, 500 mM $NaClO_4$, 3 mM $Mg(ClO_4)_2$, 5 mM buffer (pH 6.8: MOPS, pH 9.0: borate).

Materials and methods

UV measurements were performed on a CARY 100 Bio UV spectrometer using solutions containing 3 µM oligonucleotide duplex, 500 mM $NaClO_4$, 3 mM $Mg(ClO_4)_2$, and 5 mM buffer (pH 6.8: MOPS, pH 9.0: borate). UV melting curves were recorded with a heating/cooling rate of 1 °C min^{-1} and a data interval of 0.5 °C. Absorbance was normalized according to $A_{norm} = (A–A_{min})/(A_{max}–A_{min})$ at 260 nm. Melting temperatures were determined as the maxima of the first derivatives of the melting curves by applying a Gaussian fit. The two oligonucleotides were synthesized and characterized as described recently[1] (see Figures S11 and S12 for their MALDI-TOF mass spectra).

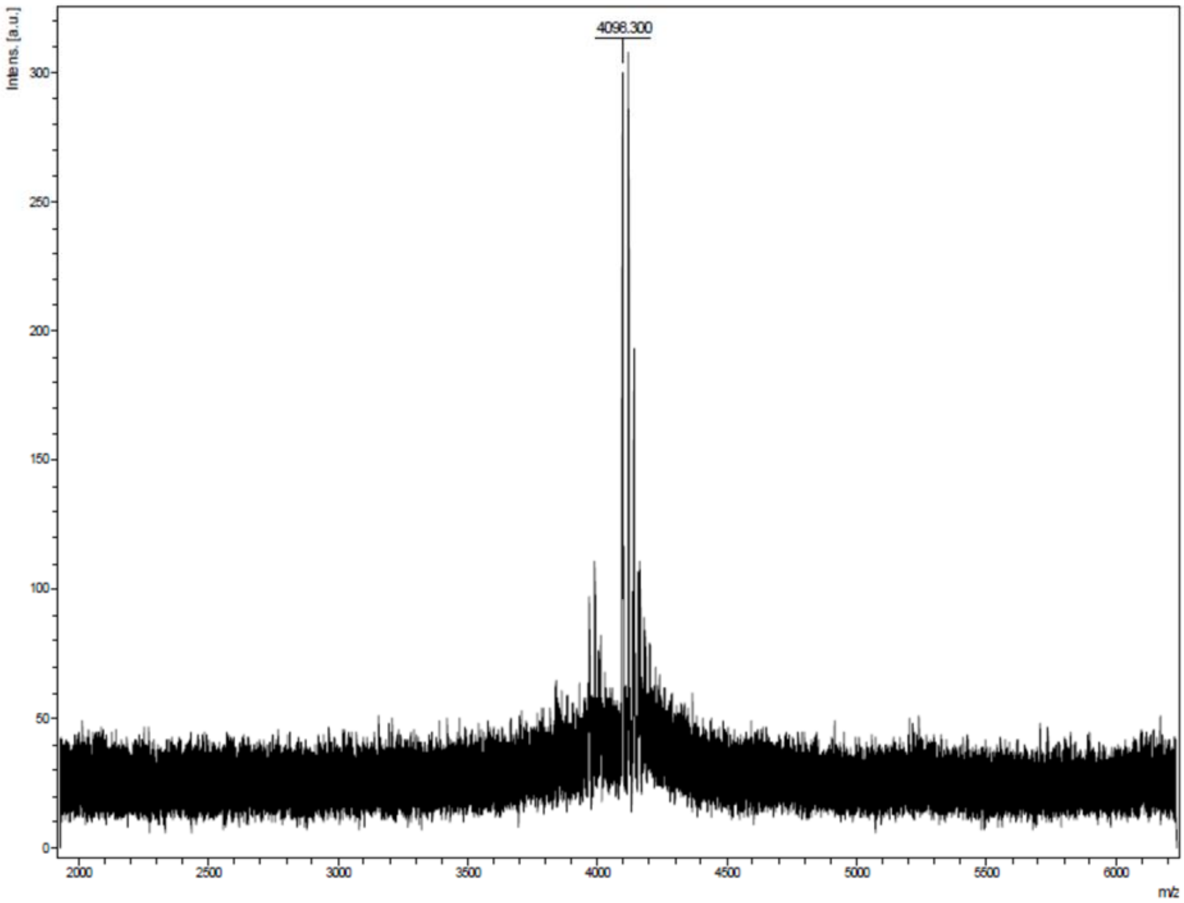

Figure S11. MALDI-TOF mass spectrum of 5′-d(GAA AGA **T**AG GGA G)-3′ ($C_{130}H_{159}N_{62}O_{71}P_{12}$, calcd. for $[M+H]^+$: 4097 Da, found: 4098 Da).

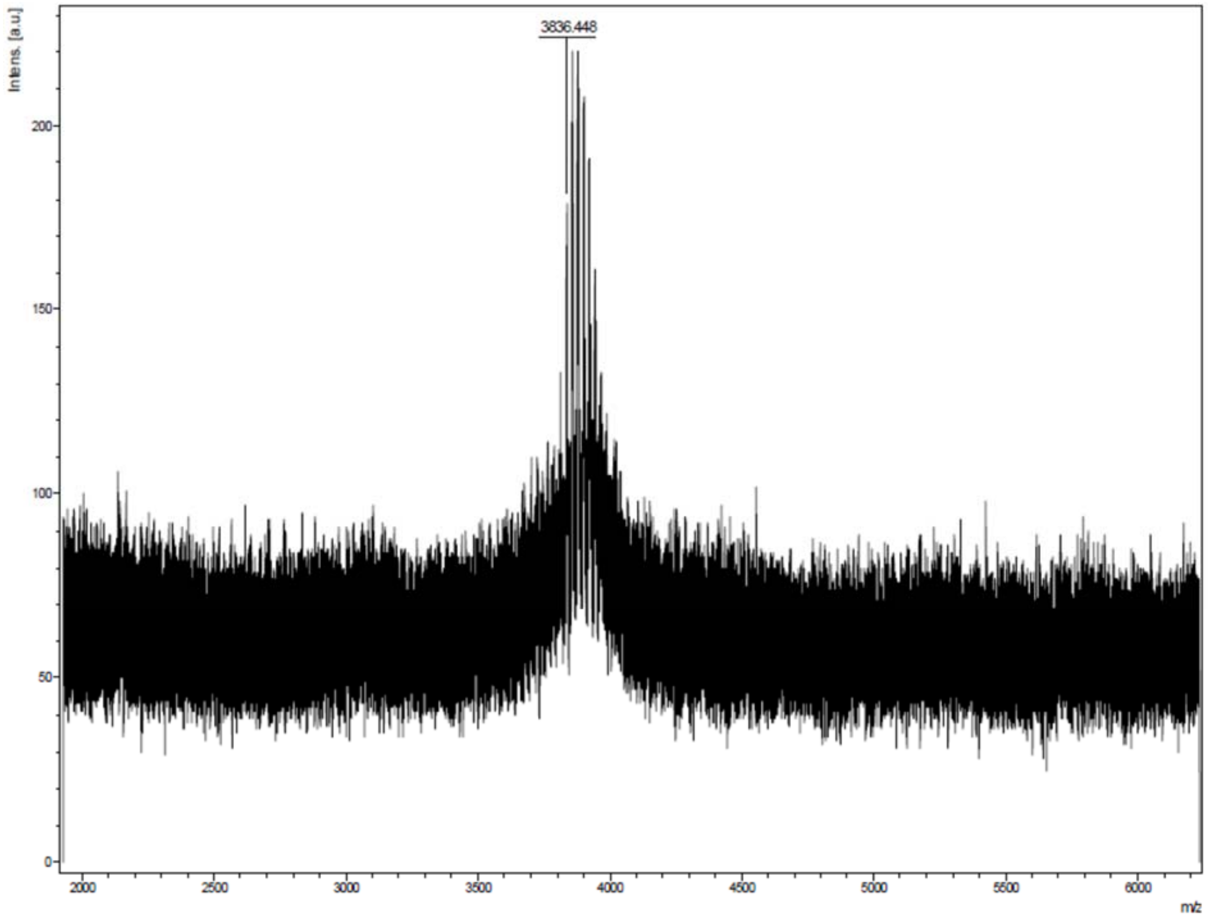

Figure S12. MALDI-TOF mass spectrum of 5′-d(CTC CCT **εA**TC TTT C)-3′ ($C_{126}H_{164}N_{35}O_{81}P_{12}$, calcd. for $[M+H]^+$: 3836 Da, found: 3836 Da).

References

1. S. Mandal, M. Hebenbrock, J. Müller, *Angew. Chem. Int. Ed.* **2016**, *55*, 15520-15523.
2. a) J. H. van de Sande, N. B. Ramsing, M. W. Germann, W. Elhorst, B. W. Kalisch, E. von Kitzing, R. T. Pon, R. C. Clegg, T. M. Jovin, *Science* **1988**, *241*, 551-557; b) V. R. Parvathy, S. R. Bhaumik, K. V. R. Chary, G. Govil, K. Liu, F. B. Howard, H. T. Miles, *Nucleic Acids Res.* **2002**, *30*, 1500-1511.
3. M. Vorlíčková, I. Kejnovská, K. Bednářová, D. Renčiuk, J. Kypr, *Chirality* **2012**, *24*, 691-698.

Files:

- DNA geometry of structure **1** (pdb) and topology for use with CP2K (inpcrd, prmtop):

dna_i_hg_wat.pdb, dna_i_hg_wat.inpcrd, dna_i_hg_wat.prmtop

- Base pair coordinates corresponding to Figs. 4 and 5:

Fig4.xyz, Fig5.xyz
